# Federated Spiking GANs with Rényi Differential Privacy for privacy-preserving synthetic EEG generation in brain–computer interfaces

**DOI:** 10.3389/fnhum.2026.1909432

**Published:** 2026-10-14

**Authors:** Shouvik Paul, Garima Bajwa

**Affiliations:** Department of Computer Science, Lakehead University, Thunder Bay, ON, Canada

**Keywords:** brain-computer interfaces, differential privacy, EEG, federated learning, Generative Adversarial Network (GAN), privacy, spiking neural network (SNN)

## Abstract

Electroencephalography (EEG) data used in brain-computer and brain-machine interfaces (BCI/BMI) contain sensitive, user-specific information, creating privacy risks in consumer applications such as gaming, human augmentation, and virtual reality. Existing methods like encryption and anonymization either fail to protect data during analysis or still expose identifiable attributes. While differential privacy (DP) offers formal protection, it often reduces data utility. The key challenge is to generate synthetic EEG data that protects sensitive attributes while retaining temporal features for downstream use. To address this, we developed a federated spiking generative framework that integrates a spiking neural network (SNN)-based generator, temporally correlated noise, an artificial neural network (ANN)-based discriminator, and Rényi differential privacy (RDP) within a federated learning framework. The SNN-based generator captures temporal characteristics, and the ANN-based discriminator maintains classification accuracy. Federated training prevents centralization of raw client data, while privacy is preserved through controlled perturbation of spike-train and membrane-potential representations, supported by RDP-based privacy accounting. We evaluated our architecture against methods such as RDP-TimeGAN, RDP-CRNN-GAN, RDP-RCGAN, and RDP-Clare-GAN, demonstrating that our approach preserves temporal features and downstream utility while maintaining privacy in synthetic EEG generation. This solution enables privacy-preserving generation of distributed synthetic EEG data for BCI/BMI research and development.

## 1 Introduction

Machine learning and neural networks have significantly impacted various fields worldwide, including medical data analysis, image processing, and natural language processing. Particularly in domains focused on data privacy and security (e.g., healthcare, neuroscience), creating synthetic data has emerged as an important area of focus. An example of this is electroencephalography (EEG) data, which capture the brain's electrical activity and are invaluable for many uses, such as medical diagnostics and emerging brain-computer interface applications. However, ensuring the confidentiality of EEG data requires robust protective measures to prevent unauthorized access or misuse.

Since Generative Adversarial Networks (GANs) can produce very high-quality, human-like data samples, they have also become a popular approach for creating synthetic data. GANs, first proposed by Goodfellow et al. (2014), are typically composed of a pair of generator and discriminator, trained jointly with the task of generating synthetic data (generator) and distinguishing real from generation-based fake data (discriminator). However, this privacy-preserving aspect of the technology remains an open issue in GAN frameworks. Researchers have explored the characteristics of machine learning models such that the synthetic data produced by the model satisfy privacy [e.g., Differential Privacy (DP), Rényi Differential Privacy (RDP)] of individuals in the original data set (Dwork et al., 2010; Mironov, 2017). These methods add noise to the data or to the model parameters, mathematically guaranteeing that individual privacy is maintained. Often, these techniques need to be integrated into GAN frameworks for synthetic data generation to produce realistic data. One of the techniques, Federated Learning (FL), has recently received considerable interest in training machine learning models on decentralized data. FL enables collaborative model training in which multiple clients can participate without revealing their data and thus help improve privacy (Paul and Bajwa, 2025).

Yan et al. (2022) empirically proved that spiking neural networks (SNN) are energy efficient and well-suited for real-time processing systems compared to classical artificial neural networks (ANN) for EMG/EEG classification, where SNN accuracies were competitive with state-of-the-art ANNs while leading to far lower power consumption. Based on this, Xu et al. (2024) used the temporal properties of SNN to capture the intricate temporal characteristics possessed by the EEG data and used them for emotion recognition. To address privacy-related issues, a GAN model, named PATE-GAN, was proposed in Jordon et al. (2019), which applies differential privacy in the generative domain using the framework of private aggregation of teacher ensembles. Although this model achieved strong privacy guarantees, it was challenging to scale and introduced additional complexity via an ensemble of teacher models, as well as the need for secure aggregation. Moreover, PATE-GAN had difficulty working with high-dimensional data because the student discriminator needs to see at least somewhat realistic generated samples from the beginning, which becomes difficult in higher-dimensional spaces. McKenna et al. with the AIM algorithm (McKenna et al., 2022), noted the importance of a balance between data utility and privacy, contributing an adaptive iterative strategy for differentially private synthetic data generation.

Esteban et al. (2017) introduced RCGAN, a recurrent conditional GAN to generate realistic time series medical data, which showed the ability of GANS to work with temporal sequences. This work paved the path for follow-up studies like Yoon et al. (2019)'s exploration, which was the first one, an implementation of TimeGAN with embedding and recovery functions to treat mixed datasets containing temporal data along with non-time-aware features. Both studies were able to process temporal data but did not include privacy-preserving technologies, creating an opportunity for additional improvement to secure data generation. Bäßler et al. extended the use of SNNs through an unsupervised anomaly detection framework over multivariate time series while focusing on its adaptive properties for online data streams (Bäßler et al., 2022). Complementing this, Khan et al. (2023) proposed a federated framework for human activity recognition, combining privacy and energy efficiency to process data from distributed edge devices. Together, these studies demonstrated the promise of using SNNs with federated learning to increase privacy and efficiency.

Nikfam et al. (2023) proposed a homomorphic encryption scheme for privacy-preserving SNN to provide strong secrecy guarantees by encrypting the input while leveraging the benefits of SNN in processing temporal data. Arsalan et al. (2022) focused on energy efficiency and privacy preservation in forecasting user health data streams using SNN to demonstrate the trade-offs between accuracy and energy savings. Rather, in the GAN world, Mogren (2016) introduced C-RNN-GAN to adversarial training from continuous sequential data such as music generation. This paper showed that these GANs have the potential to process temporal data. In this sense, Rosenfeld et al. (2022) extended the concept with Spiking GANs to incorporate local training and Bayesian models, as well as continuous meta-learning to enhance adaptability and efficiency. This research underscored that continued advancements in GANs and SNN led to more efficient generation of synthetic data examples.

Shen et al. (2025) introduced temporal spiking GANs for heading direction decoding, a form of EEG signal processing, and showed the importance of temporal dynamics in this research stream. Similarly, Wang et al. (2022) proposed DPSNN to address the differential privacy of temporal data processing by using temporal enhanced pooling to improve utility and robustness with respect to differentially private training. These experiments showcased the power of privacy-preserving mechanisms when coupled with GANs while dealing particularly with temporal data. Furthermore, the concept of PPGAN by Liu et al. (2019) deliberately combines differential privacy with GANs by adding noise to the gradient during generator training. Later, Xie et al. (2018) extended this work as DPGAN, introducing differential privacy in data generation through noise addition, demonstrating that both privacy and the usefulness of the generated data are crucial.

Although synthetic data generation and privacy-preserving tools have advanced significantly recently, some notable limitations remain. Numerous studies highlight the considerable potential of employing SNNs to process EEG data (Yan et al., 2022; Xu et al., 2024), yet none have successfully provided a comprehensive approach to preserving all forms of private information. For example, PATE-GANs have been developed to address privacy concerns but face challenges with scalability and high-dimensional data (Jordon et al., 2019). Furthermore, despite progress in the generation of temporal data with GANs (Esteban et al., 2017; Yoon et al., 2019), these methods lack the incorporation of differential privacy. Several studies examined the self-adaptiveness of SNNs and federated frameworks for tasks such as anomaly detection (AD) and human activity recognition (Bäßler et al., 2022; Khan et al., 2023), but indicated the need for privacy-preserving mechanisms. Recent studies have further explored deep-learning-based EEG generation and analysis, including biologically informed EEG simulation for synthetic dataset generation (Bunterngchit et al., 2025a; Paul and Bajwa, 2026) and explainable deep-learning approaches for EEG-based neurological disease detection (Bunterngchit et al., 2025b).

Various studies have been conducted on privacy-preserving techniques for SNNs, for example, in homomorphic encryption and energy efficiency; however, this area of research typically does not offer direct applications to EEG data or include advanced temporal coding (Nikfam et al., 2023; Arsalan et al., 2022). Similarly, continuous sequential data GANs, including Spiking GANs, have reported significant progress in data generation quality, but do not address privacy concerns (Mogren, 2016; Rosenfeld et al., 2022). Recent studies on GANs, equipped with temporal dynamics and differential privacy mechanism, showcase effectiveness while highlighting the need for further integration of federated learning and advanced temporal coding (Shen et al., 2025; Wang et al., 2022; Paul, 2024).

In this study, we propose a Federated Spiking GAN framework that integrates spiking neural networks (SNNs) with Generative Adversarial Networks (GANs), coupled with an advanced noise model for the synthesis of EEG data. Our approach exploits the temporal dynamics of SNNs to produce more realistic and temporally coherent synthetic data, while the ANN-based discriminator ensures classification accuracy. We employ a temporally correlated noise model with Rényi Differential Privacy (RDP) to provide a reliable privacy guarantee alongside high-quality data utility. By addressing privacy and utility issues jointly, our work advances the domain of privacy-preserving synthetic data generation for sensitive EEG data. We introduce a federated learning architecture to enhance data privacy, where model training occurs in a decentralized manner across client nodes without exchanging raw data, providing additional model robustness. Our comprehensive assessments evaluate the trade-offs between privacy and data utility, validating the effectiveness of our framework.

EEG signals are widely used in brain-computer interfaces, neurological assessment, cognitive-state analysis, and machine-learning-based biomedical research. However, because EEG recordings contain subject-specific physiological information, sharing them across institutions is challenging. This creates a conflict between the need for large datasets and the requirement to protect participant privacy. The proposed framework is intended for synthetic data generation, not for altering clinically acquired EEG used in diagnosis. The generated EEG supports BCI algorithm development, data augmentation, model pre-training, benchmarking, and collaborative machine-learning research where sharing raw EEG is not feasible. Specifically, synthetic motor-imagery EEG can supplement training datasets for BCI classifiers in applications such as authentication, neurogaming, and wheelchair control, without exposing individual recordings.

The proposed framework is also applicable to federated research environments in which several institutions possess EEG data but cannot transfer the original recordings to a central location. In such a setting, the local models are trained using locally available EEG data, while only model information is exchanged through the federated-learning procedure. Importantly, this method is not intended to add arbitrary noise to EEG used for clinical diagnosis. Privacy protection is applied during learning and synthetic data generation, and the resulting data are intended for research, algorithm development, augmentation, and evaluation. Clinical use would require further validation with clinically representative datasets and adherence to regulatory procedures. Mobini et al. (2026) address secure EEG transmission with authorized recovery of the original signal. In contrast, our framework generates privacy-preserving synthetic EEG data, aiming to produce task-related samples without exposing or enabling reconstruction of the original recordings. Therefore, while Mobini et al. (2026) enable authorized recovery, our framework prioritizes preventing recovery of the original EEG as a key privacy objective.

## 2 Materials and methods

### 2.1 Preliminaries

This section introduces the theoretical and methodological foundations required to describe the proposed privacy-preserving federated SNN-GAN framework. First, the fundamental definition of differential privacy is presented to establish the formal notion of privacy used throughout the work. Next, Rényi differential privacy is introduced because it provides a convenient framework for accumulating privacy loss over the repeated randomized operations involved in DP-SGD. The gradient clipping and Gaussian perturbation equations are then presented to describe how the formal DP mechanism is implemented during discriminator training. These preliminaries provide the basis for the privacy-preserving training procedure and the RDP-based privacy accounting used in the proposed framework.

#### 2.1.1 Privacy-preserving mechanisms

The privacy-preserving mechanisms used in this work are introduced to establish the mathematical basis for protecting EEG samples during model training and to quantify the resulting privacy guarantee. First, differential privacy (DP) provides the formal definition of privacy with respect to changes in an individual data record. Second, Rényi differential privacy (RDP) provides a convenient representation for composing the privacy loss over repeated randomized training operations. Finally, the gradient perturbation mechanism is used to implement DP during discriminator training through per-example gradient clipping and Gaussian noise addition. These formulations are therefore stated to define the privacy guarantee, provide the RDP representation used for privacy accounting, and describe the DP-SGD update actually applied in the proposed federated training framework.

Differential Privacy (DP) is one of the most popular mathematical frameworks for quantifying privacy guarantees (Dwork et al., 2010). This is done by introducing controlled noise into the data or model parameters so that adding/removing an individual data point cannot change the output, thereby protecting individual privacy.

Differential privacy (DP) is a well-founded algorithmic framework that guarantees that individual data is private while simultaneously allowing the use of such information for useful data analysis. If an observer cannot determine whether information belonging to a given individual is included in the input dataset, then the function is considered differentially private.

A randomized function F is considered ϵ-differentially private if, for any pair of datasets X and Y differing by only one element, and for any possible output subset Z, the condition in Equation 1 is satisfied:


$$\begin{array}{l} \mathrm{Pr}\left[F\left(X\right)\in Z\right]\leq e^{\epsilon }\mathrm{Pr}\left[F\left(Y\right)\in Z\right] \end{array}$$ (1)

Here, ϵ is a parameter that controls the level of privacy, with smaller values of ϵ providing stronger privacy guarantees. This definition safeguards the privacy of individual data points by ensuring that the algorithm's output distribution does not change significantly when any data point in the input dataset is changed.

Rényi divergence is used to quantify privacy loss in Rényi Differential Privacy (RDP), an extension of DP (Mironov, 2017). This approach provides a more flexible and refined privacy analysis. For a randomized function H and datasets X and Y differing in one element, RDP is defined for an order α>1 according to Equation 2:


$$\begin{array}{l} D_{\alpha }\left(H\left(X\right)|H\left(Y\right)\right)=\frac{1}{\alpha -1}\log 𝔼\left[{\left(\frac{\mathrm{Pr}\left[H\left(X\right)=\omega \right]}{\mathrm{Pr}\left[H\left(Y\right)=\omega \right]}\right)}^{\alpha }\right] \end{array}$$ (2)

A mechanism H satisfies (α, ϵ)-RDP if it fulfills Equation 3:


$$\begin{array}{l} D_{\alpha }\left(H\left(X\right)|H\left(Y\right)\right)\leq \epsilon \end{array}$$ (3)

where, the Renyi divergence order is denoted by α and the privacy budget by ϵ. Tighter privacy assurances are possible under specific circumstances due to RDP's more flexible and sophisticated approach to privacy analysis.

Abadi et al. (2016) presented a technique that adds noise to gradients during training to enable DP in deep learning. This approach guarantees differential privacy during the learning phase. The differentially private gradient descent involves clipping the gradients and adding Gaussian noise. Let **g**_*t*_ represent the gradient at the *t*-th iteration, then the clipped gradient ${\tilde{\text{g}}}_{t}$ is defined by Equation 4:


$$\begin{array}{l} {\tilde{\text{g}}}_{t}=\frac{{\text{g}}_{t}}{\max\left(1,\frac{|{\text{g}}_{t}{|}_{2}}{\gamma }\right)} \end{array}$$ (4)

where γ is the clipping threshold. Gaussian noise $N\left(0,\sigma^{2}\gamma^{2}\right)$ is then added to the clipped gradients as described in Equation 5:


$$\begin{array}{l} {\hat{\text{g}}}_{t}={\tilde{\text{g}}}_{t}+N\left(0,\sigma^{2}\gamma^{2}\right) \end{array}$$ (5)

The updated model parameters **θ** are computed as shown in Equation 6:


$$\begin{array}{l} \theta_{t+1}=\theta_{t}-\eta {\hat{\text{g}}}_{t} \end{array}$$ (6)

where η is the learning rate. This method ensures that the model's parameters remain differentially private while allowing the model to learn effectively.

In the proposed framework, the gradient-based DP mechanism is applied to the discriminator during local client training. For each discriminator update, gradients are first computed for individual EEG examples and clipped to the prescribed ℓ_2_-norm bound. Gaussian noise, controlled by the DP noise multiplier σ_DP_, is then added to the aggregated clipped gradients before the discriminator parameters are updated. The resulting discriminator update is therefore the formal DP mechanism used for privacy accounting.

Phan et al. (2017) proposed the Adaptive Laplace Mechanism, which enhances DP by injecting noise adaptively based on the relevance of model parameters to the output. This mechanism adjusts the amount of noise added to each parameter, balancing privacy and utility. For a model parameter θ_*i*_, the noise is sampled from a Laplace distribution with scale β_*i*_ according to Equation 7:


$$\begin{array}{l} L\left(0,\beta_{i}\right)=\frac{1}{2\beta_{i}}\exp\left(-\frac{\left|x\right|}{\beta_{i}}\right) \end{array}$$ (7)

The adaptive mechanism adjusts β_*i*_ based on the sensitivity of θ_*i*_ to the input data, ensuring that the less relevant parameters receive more noise, while the more relevant parameters receive less noise. The updated parameter $\theta_{i}^{\prime }$ is given by Equation 8:


$$\begin{array}{l} \theta_{i}^{\prime }=\theta_{i}+L\left(0,\beta_{i}\right) \end{array}$$ (8)

For the proposed federated SNN-GAN, the Laplace mechanism is presented as a related privacy mechanism but is not used for the formal privacy accounting of the reported experiments. The reported privacy budget is obtained from the Gaussian perturbation applied to the clipped discriminator gradients and accumulated using RDP.

#### 2.1.2 Generative Adversarial Networks (GANs)

Generative Adversarial Networks (GANs), first proposed in Goodfellow et al. (2014), are a powerful family of machine learning models. A discriminator (*D*) and a generator (*G*) are the two neural networks that make up a GAN. The first network, *G*, generates fake data from noise. The second is a discriminator *D* that is trained to distinguish between real and synthetic data. This adversarial process causes *G* to create higher-quality data. GANs' goal may be expressed as a minimax game according to Equation 9:


$$\begin{array}{r} \underset{G}{\min}\underset{D}{\max}V\left(D,G\right)={𝔼}_{\text{x}\sim p_{\text{data}}\left(\text{x}\right)}\left[\log D\left(\text{x}\right)\right]\\ +{𝔼}_{\text{z}\sim p_{\text{z}}\left(\text{z}\right)}\left[\log\left(1-D\left(G\left(\text{z}\right)\right)\right)\right] \end{array}$$ (9)

Both the (*G*) and (*D*) networks in traditional GANs are implemented by Convolutional Neural Networks (CNN). The generator continues this process by transforming noise into synthetic data through convolutional layers, while the discriminator labels real or fake data using similar layers. This method works well for problems requiring continuous data representations, such as generating images. In contrast to these approaches, Spiking GANs employ SNNs in the generator and discriminator. SNNs' spiking architecture, which processes data as discrete spikes, makes it especially useful for monitoring temporal patterns like those seen in EEG signals. The SNN-based *G* generates synthetic spike patterns, while the SNN-based *D* compares these patterns with real spike data.

#### 2.1.3 Federated learning

With Federated Learning (FL), several clients can train together to build a common model without sharing their raw data due to a decentralized ML technique (Li et al., 2020). FL protects the privacy of user data by keeping data on local servers, reducing the possibility of security breaches, and guaranteeing adherence to data protection laws. FL uses a variety of distributed datasets to improve the generalizability of models (McMahan et al., 2017).

In FL, each client trains locally on their own private data and only shares updates to the model with a central server (which aggregates these updates to update the global model). The federated averaging algorithm that is at the heart of FL is defined as by Equation 10:


$$\begin{array}{l} {\text{w}}_{t+1}={\text{w}}_{t}-\eta \overset{N}{\underset{i=1}{\sum }}\frac{n_{i}}{n}{\text{u}}_{i}^{t} \end{array}$$ (10)

where **w**_*t*_ represents the model parameters in round *t*, η is the learning rate, ${\text{u}}_{i}^{t}$ is the update from client *i*, *n*_*i*_ is the number of samples held by client *i*, and *n* is the total number of samples between all clients.

Federated Learning (FL) addresses various issues including high communication expenses, data variability or heterogeneity, and security. Beyond healthcare, Liu et al. (2020) have investigated applications utilizing FL in areas like privacy-focused traffic flow prediction. Mothukuri et al. (2021) conducted a comprehensive survey on the security and privacy challenges in FL, emphasizing the necessity of incorporating robust privacy-preserving mechanisms into FL systems.

### 2.2 Methodology

Our proposed methodology introduces an advanced GAN framework for generating privacy-preserving synthetic EEG data. This framework uniquely integrates a spiking neural network (SNN)-based generator with an artificial neural network (ANN)-based discriminator. Our model excels at producing high-fidelity synthetic EEG signals that capture the temporal dynamics of SNNs while maintaining the computational efficiency of ANNs. The spiking neurons within the generator simulate the behavior of biological neurons, while the discriminator's multi-layered ANN architecture is optimized to assess the authenticity of the generated data.

The architecture of the GAN comprises several layers in both the generator and discriminator, each designed to play a critical role in processing and capturing the complex patterns in EEG signals. The SNN-based generator is built on a modified Leaky Integrate-and-Fire (LIF) neuron model, which is well-suited to capturing the temporal dynamics of EEG signals. The ANN-based discriminator complements this by efficiently distinguishing real from synthetic EEG data using a series of fully connected layers. Our methodology is shown in Figure 1 and is detailed in Algorithm 1. The internal components of the local client is shown in Figure 2.

**Figure 1:**
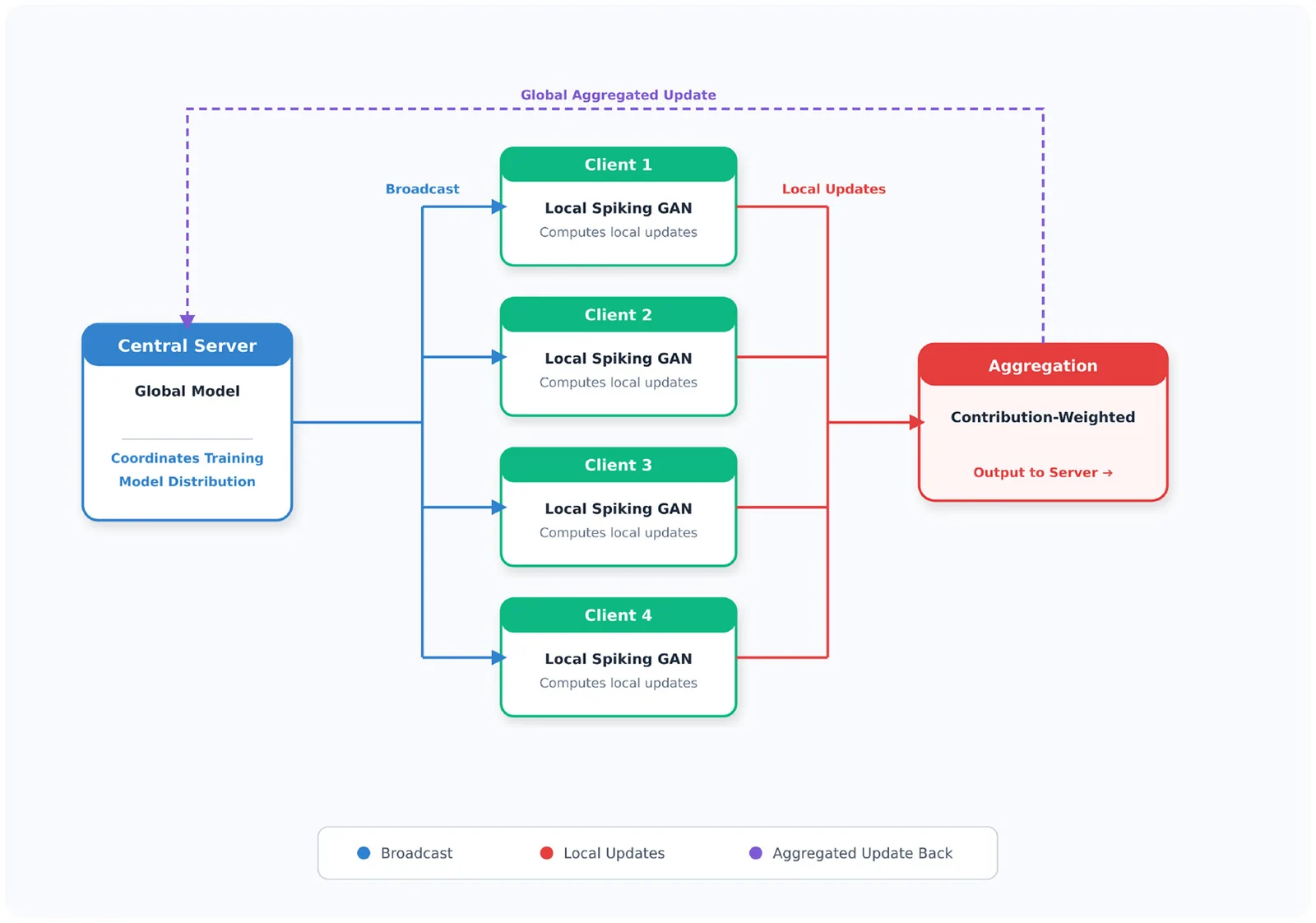
Schematic workflow of the proposed Federated Spiking GAN method.

**Algorithm 1:**
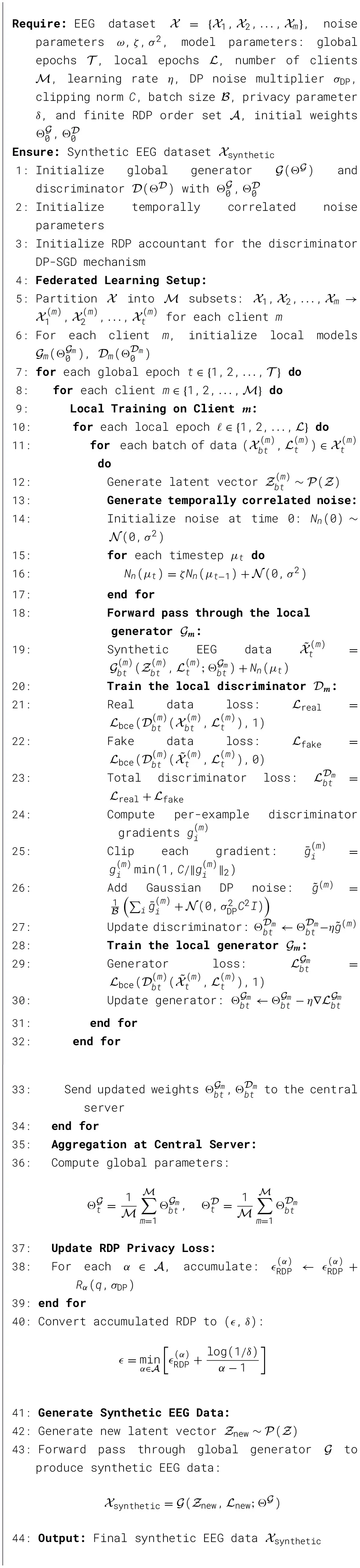
Privacy-preserving synthetic EEG data generation using Federated Spiking GAN.

**Figure 2:**
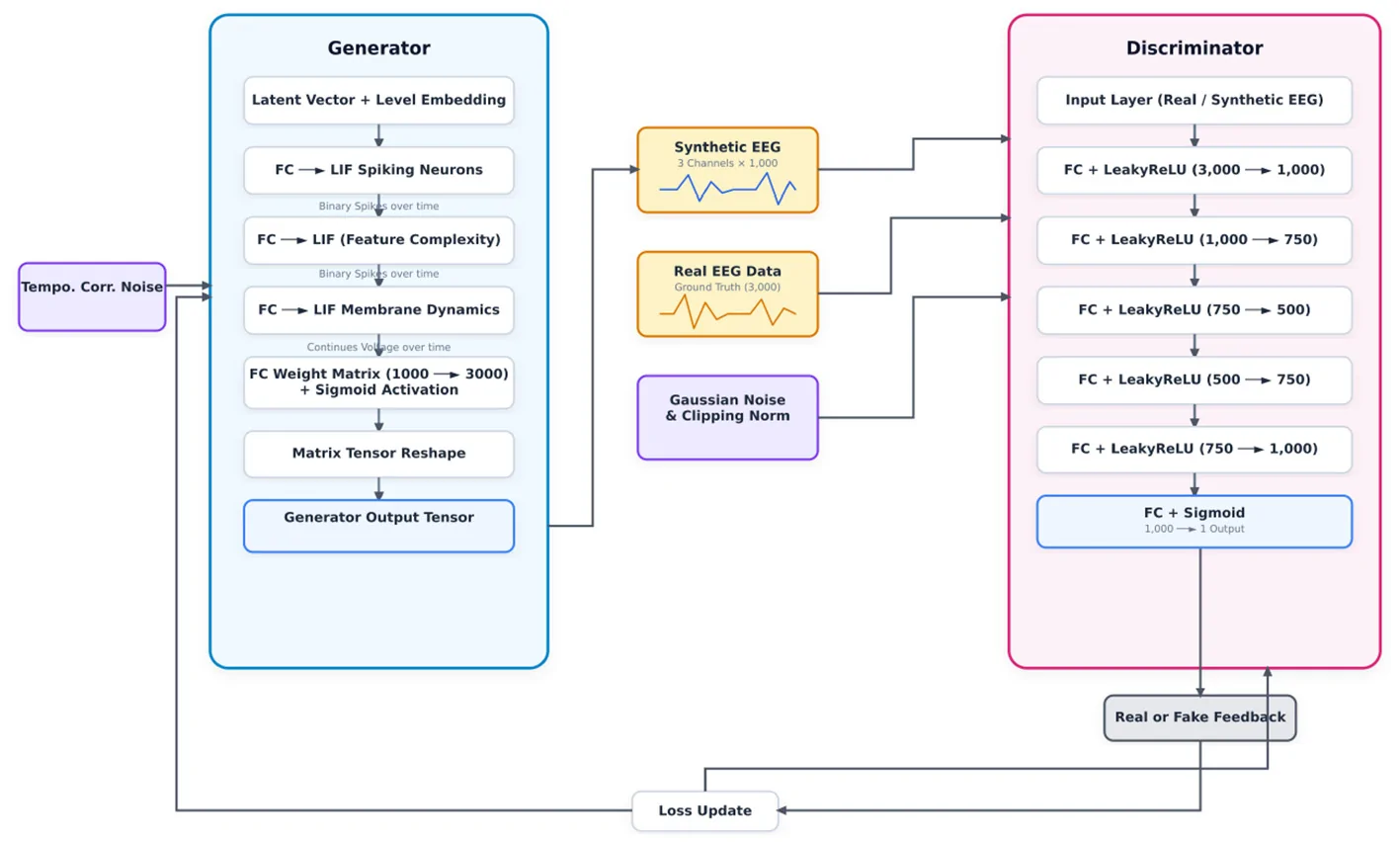
Schematic diagram of the local client module, including the spiking neural network components and local discriminator-generator setup.

We developed a federated learning framework that enables multiple clients to independently train Spiking GAN models using their own private EEG datasets. This methodology guarantees the decentralization of sensitive EEG data, safeguards privacy, and facilitates the improvement of collaborative models. At the beginning of every communication round, the server sends the global model parameters to each client. Then, using spike-timing-dependent learning algorithms and temporally correlated noise mechanisms to mimic neuronal spiking behavior, the client processes the EEG data by training a local Spiking GAN model. Sensitive information is kept localized because, upon completion of the local training, the client sends the updated model's parameters (gradients) back to the server without releasing any raw EEG data.

The fundamental component of this framework, the server, is responsible for combining the local updates from every client. Without accessing any client's raw data, the server uses Federated Averaging to merge the locally updated model parameters into a single global model. Over several rounds, this method iteratively improves model performance, with each communication cycle improving model performance. To provide a formal differential privacy guarantee for the real EEG data accessed during training, the discriminator is trained using a differentially private stochastic gradient descent (DP-SGD) mechanism. In particular, per-example discriminator gradients are clipped to a fixed ℓ_2_ norm and Gaussian noise is added to the aggregated clipped gradients before updating the discriminator parameters. Rényi Differential Privacy (RDP) is then used to account for the cumulative privacy loss over the sequence of DP-SGD updates. The temporally correlated noise used in the SNN generator is treated separately from the formal DP mechanism because its samples are temporally dependent and therefore are not composed as independent Gaussian mechanisms in the RDP accountant.

#### 2.2.1 Data generation process

The data generation process in our framework comprises several stages, each crucial to ensuring that the synthetic EEG data produced is realistic and preserves privacy. These stages begin with the theoretical foundation and model design, which are deeply rooted in the principles of spiking neural networks. In contrast to traditional neural networks that rely on continuous activation functions, spiking neurons operate based on discrete events–spikes. This discrete nature allows SNNs to capture the temporal dynamics inherent in EEG signals more effectively.

#### 2.2.2 Membrane potential dynamics of the neurons

The neurons in our generator are modeled using a modified version of the Leaky Integrate-and-Fire (LIF) model (Gao et al., 2023; Singanamalla, 2022). The membrane potential *u*_*i*_ of the neuron *i* at any time $\bar{t}$ is described by Equation 11:


$$\begin{array}{l} \tau_{m}\frac{du_{i}}{d\bar{t}}=-\left(u_{i}-u_{\text{rest}}\right)+RI_{i}\left(\bar{t}\right) \end{array}$$ (11)

where, τ_*m*_ is the membrane time constant, *u*_rest_ is the resting potential, *R* is the input resistance, and $I_{i}\left(\bar{t}\right)$ is the input current.

When the membrane potential *u*_*i*_ reaches a threshold ϑ, the neuron fires a spike, and the potential is reset to *u*_rest_. The spike generation and reset mechanism are incorporated through the spike train $S_{i}\left(\bar{t}\right)$, leading to the relationship described in Equation 12 (Singanamalla, 2022):


$$\begin{array}{l} \tau_{m}\frac{du_{i}}{d\bar{t}}=-\left(u_{i}-u_{\text{rest}}\right)+RI_{i}\left(\bar{t}\right)+S_{i}\left(\bar{t}\right)\left(u_{\text{rest}}-\vartheta \right) \end{array}$$ (12)

where, the spike train $S_{i}\left(\bar{t}\right)$ is defined by Equation 13:


$$\begin{array}{l} S_{i}\left(\bar{t}\right)=\underset{k}{\sum }\delta \left(\bar{t}-{\bar{t}}_{k}^{i}\right) \end{array}$$ (13)

where, ${\bar{t}}_{i}^{k}$ represents the *k*-th spike time of neuron *i*.

#### 2.2.3 Discretization of membrane potential for implementation

To implement the neuron dynamics in discrete time, we approximate the continuous-time differential equation using small time steps $\Delta \bar{t}$. The discretized update rule for the membrane potential at time step *q* is given by Singanamalla (2022) in Equation 14:


$$\begin{array}{l} u_{i}\left[q+1\right]=\tilde{\beta }u_{i}\left[q\right]+I_{i}\left[q\right]-S_{i}\left[q\right] \end{array}$$ (14)

where, $\tilde{\beta }\equiv \exp\left(-\frac{\Delta \bar{t}}{\tau_{m}}\right)$, *S*_*i*_[*q*] represents the spike at the *q*-th time step, and *S*_*i*_[*q*] = 1 (if spike), *S*_*i*_[*q*] = 0 (if no spike).

The input current *I*_*i*_[*q*] is updated similarly according to Equation 15:


$$\begin{array}{l} I_{i}\left[q+1\right]=\tilde{\alpha }I_{i}\left[q\right]+\underset{j}{\sum }W_{ij}S_{j}\left[q\right] \end{array}$$ (15)

where, $\tilde{\alpha }\equiv \exp\left(-\frac{\Delta \bar{t}}{\tau_{s}}\right)$, and *W*_*ij*_ is the synaptic weight between neuron *j* and neuron *i*.

#### 2.2.4 Spike generation and membrane reset

When the membrane potential *u*_*i*_ reaches the threshold ϑ, the neuron generates a spike, modeled by the Dirac delta function $\delta \left(\bar{t}-{\bar{t}}_{k}^{i}\right)$, and the potential is reset to *u*_rest_. The spike generation and reset process is represented in discrete time by Equation 16:


$$\begin{array}{l} S_{i}\left[q\right]=\{\begin{array}{cc} 1, & u_{i}\left[q\right]\geq \vartheta ,\\ 0, & u_{i}\left[q\right]<\vartheta , \end{array} \end{array}$$ (16)

and the membrane reset is updated according to Equation 17


$$\begin{array}{l} u_{i}^{\text{reset}}\left[q\right]=\left(1-S_{i}\left[q\right]\right)u_{i}\left[q\right]+S_{i}\left[q\right]u_{\text{rest}} \end{array}$$ (17)

where *S*_*i*_[*q*] denotes the binary spike generated at time step *q*, ϑ is the firing threshold, and *u*_rest_ is the reset potential. Thus, when *u*_*i*_[*q*]≥ϑ, the neuron emits a spike and its membrane potential is reset to *u*_rest_; otherwise, the membrane potential follows the discretized membrane dynamics. This explicitly defines the discrete thresholding and reset operation used in the spiking generator.

#### 2.2.5 Synaptic filtering for spike trains

To convert discrete spike trains into biologically plausible continuous signals, a double exponential synaptic filter (Nicola and Clopath, 2017; Singanamalla, 2022) is applied. The filtered response *r*_*j*_ of neuron *j* is governed by by the system of equations defined in Equations 18 and 19:


$$\begin{array}{l} \dot{r_{j}}=-\frac{r_{j}}{\tau_{d}}+h_{j} \end{array}$$ (18)

$$\begin{array}{l} \dot{h_{j}}=-\frac{h_{j}}{\tau_{r}}+\frac{1}{\tau_{r}\tau_{d}}\underset{{\bar{t}}_{j}<\bar{t}}{\sum }\delta \left(\bar{t}-{\bar{t}}_{j}^{k}\right) \end{array}$$ (19)

where, τ_*r*_ and τ_*d*_ are the synaptic rise and decay time constants, *h*_*j*_ is an intermediary signal, $\delta \left(\bar{t}-{\bar{t}}_{j}^{k}\right)$ is the spike train of neuron *j*.

This synaptic filtering process produces continuous signals from discrete spikes, mimicking the biological processes of real neurons.

#### 2.2.6 Privacy preservation using temporally correlated noise

To maintain the temporal variability of the generated EEG data, temporally correlated noise is introduced into the dynamics of the membrane potential during the training process. This perturbation is distinguished from the formal differential privacy mechanism described later in this section. In particular, the temporally correlated noise is introduced within the SNN generator to influence its temporal dynamics and generation process, whereas the formal privacy guarantee for real EEG data is provided by DP-SGD applied to the discriminator gradients.

The noise Noise_*n*_(μ) for neuron *n* is modeled as Equation 20:


$$\begin{array}{l} {\text{Noise}}_{n}\left(\bar{t}\right)=\zeta \cdot {\text{Noise}}_{n}\left(\bar{t}-\Delta \bar{t}\right)+\xi \left(\bar{t}\right) \end{array}$$ (20)

where, ζ is the correlation coefficient determining the degree of temporal correlation in the noise, and $\xi \left(\bar{t}\right)\sim N\left(0,\sigma^{2}\right)$ is Gaussian noise with mean 0 and variance σ^2^.

This noise is then incorporated into the membrane potential equation as Equation 21:


$$\begin{array}{l} u_{n}\left(\bar{t}+\Delta \bar{t}\right)=\tilde{\beta }u_{n}\left(\bar{t}\right)+I_{n}\left(\bar{t}\right)-S_{n}\left(\bar{t}\right)+{\text{Noise}}_{n}\left(\bar{t}\right) \end{array}$$ (21)

The temporally correlated perturbation is used to modify the stochastic dynamics of the generator and to investigate its effect on the temporal characteristics and utility of the generated EEG. Because the noise is autoregressive, consecutive noise values are statistically dependent. Therefore, these perturbations are not treated as a sequence of independent Gaussian mechanisms in the RDP accountant. The formal privacy budget is instead determined by the DP-SGD mechanism applied to the discriminator, as described below.

#### 2.2.7 Federated learning and model aggregation

In this work, we implemented a federated learning framework by simulating both client-side and server-side processes on a single personal laptop. Although federated learning typically involves separate devices, we replicated the federated environment entirely on one machine for practical purposes. The dataset was partitioned between four simulated clients, each receiving an equal portion of the data. Each client initialized local instances of the SNN-based generator and ANN-based discriminator models, synchronized with the global model at the start of each training round. Clients trained their local models for four local epochs, processing data in mini-batches of size 8. After training, each client computed its contribution to the global model by evaluating the performance of its local discriminator and quantifying it using a function that measures the difference between real and generated spike-train outputs.

Let *c*_*m*_ denote the contribution score obtained by client *m* from the prescribed local discriminator evaluation. The contribution scores are normalized across the participating clients to obtain the aggregation weights:


$$\begin{array}{l} w_{m}=\frac{c_{m}}{\sum_{j=1}^{M}c_{j}},\;\overset{M}{\underset{m=1}{\sum }}w_{m}=1, \end{array}$$ (22)

where *c*_*m*_ represents the measured contribution of client *m*, *w*_*m*_ is its corresponding aggregation weight, and M=4 is the number of participating clients. Thus, clients with larger contribution scores have proportionally greater influence on the aggregated global model.

Consequently, the number of complete mini-batches in a local epoch for client *m* is $\left\lfloor N_{m}/B\right\rfloor$, where *N*_*m*_ denotes the number of samples available to client *m*. The remaining samples that do not form a complete mini-batch are not used in that epoch. This procedure was applied consistently across subjects after artifact removal.

The server-side aggregation was simulated on the same machine. After receiving updates from all clients, we used contribution-weighted aggregation to update the global model, ensuring that clients with higher contributions had a greater impact. The contribution-weighted aggregation of the generator and discriminator parameters is therefore expressed as shown in Equation 23:


$$\begin{array}{l} \Theta_{t}^{G}=\overset{M}{\underset{m=1}{\sum }}w_{m}\Theta_{bt}^{G_{m}},\;\Theta_{t}^{D}=\overset{M}{\underset{m=1}{\sum }}w_{m}\Theta_{bt}^{D_{m}} \end{array}$$ (23)

The formal privacy accounting was performed over the actual number of discriminator DP-SGD updates rather than using the number of local epochs alone. For each client *m*, the number of discriminator updates is determined from its local dataset size, batch size, local epochs, and number of communication rounds.

To maintain privacy, we incorporated Rényi Differential Privacy (RDP) and calculated the privacy budget from the cumulative DP-SGD steps, as described in the next subsection.

In our federated learning setup, the EEG dataset $X=\left\{X_{1},X_{2},\ldots ,X_{m}\right\}$ was partitioned into multiple subsets among clients *m*, ensuring that each client only has access to its own local data (Li et al., 2020). This decentralized structure improves privacy by keeping the raw EEG data localized, and only model updates are communicated to the central server. In the beginning, the global generator G and discriminator D were initialized on the central server, with initial weights $\Theta_{0}^{G}$ and $\Theta_{0}^{D}$. The noise parameters ζ and σ^2^ were applied during the generation of synthetic EEG data to introduce stochastic temporal dynamics.

Each client trains its own local copy of the generator $G_{m}$ and discriminator $D_{m}$ using its local subset $X_{t}^{\left(m\right)}$ of the EEG dataset. During local training, the generator produces synthetic EEG data by sampling latent vectors Z from a predefined distribution P(Z). At the same time, the local discriminator $D_{m}$ was trained to differentiate between real and synthetic EEG data. Real $L_{\text{real}}$ and fake EEG data losses $L_{\text{fake}}$ were computed using binary cross-entropy loss functions. The discriminator was updated using gradient descent to minimize these loss functions, allowing it to improve its ability to classify real and synthetic data. The real and fake losses are given by Ho and Wookey (2019), Li et al. (2022), and Tabassum et al. (2022) in Equations 24 and 25, respectively:


$$\begin{array}{l} L_{\text{real}}=L_{\text{bce}}\left(D_{bt}^{\left(m\right)}\left(X_{bt}^{\left(m\right)}\right),1\right) \end{array}$$ (24)

$$\begin{array}{l} L_{\text{fake}}=L_{\text{bce}}\left(D_{bt}^{\left(m\right)}\left({\tilde{X}}_{t}^{\left(m\right)}\right),0\right) \end{array}$$ (25)

For the discriminator update, the loss is evaluated on individual examples in the mini-batch to obtain per-example gradients. Let $g_{i}^{\left(m\right)}$ denote the discriminator gradient associated with example *i* on client *m*. Each gradient is clipped to the maximum ℓ_2_ norm *C* as


$$\begin{array}{l} {ḡ}_{i}^{\left(m\right)}=g_{i}^{\left(m\right)}\min\left(1,\frac{C}{\|g_{i}^{\left(m\right)}{\|}_{2}}\right) \end{array}$$ (26)

where *C* = 1.0 is the clipping norm. The clipped gradients are aggregated and Gaussian noise is added before the discriminator update:


$$\begin{array}{l} {\tilde{g}}^{\left(m\right)}=\frac{1}{B}\left(\underset{i\in B}{\sum }{ḡ}_{i}^{\left(m\right)}+N\left(0,\sigma_{\text{DP}}^{2}C^{2}I\right)\right) \end{array}$$ (27)

where σ_DP_ denotes the DP noise multiplier, *C* is the clipping norm, B is the mini-batch size, and *I* is the identity matrix. The discriminator parameters are then updated according to


$$\begin{array}{l} \Theta_{bt}^{D_{m}}\leftarrow \Theta_{bt}^{D_{m}}-\eta {\tilde{g}}^{\left(m\right)} \end{array}$$ (28)

After training the discriminator, the generator was updated using the discriminator's feedback. The generator's goal is to improve the realism of its generated EEG data so that the discriminator cannot easily distinguish between real and synthetic data. The generator loss was calculated similarly using binary cross-entropy loss, defined by Equation 29:


$$\begin{array}{l} L_{bt}^{G_{m}}=L_{\text{bce}}\left(D_{bt}^{\left(m\right)}\left({\tilde{X}}_{t}^{\left(m\right)},L_{t}^{\left(m\right)}\right),1\right) \end{array}$$ (29)

The generator update uses the discriminator's privacy-preserving output and does not directly access the real EEG batch. Thus, the generator parameters and generated samples are treated as post-processing of the information available through the DP-protected discriminator training, expressed as Equation 30:


$$\begin{array}{l} \Theta_{bt}^{G_{m}}\leftarrow \Theta_{bt}^{G_{m}}-\eta \nabla_{\Theta^{G_{m}}}L_{bt}^{G_{m}} \end{array}$$ (30)

Once local training was completed, the updated model weights $\Theta_{bt}^{G_{m}}$ and $\Theta_{bt}^{D_{m}}$ were sent to the central server, where they were aggregated.

Using the contribution weights defined in Equation 22, the server performs weighted model aggregation rather than uniform averaging, as formalized in Equation 31:


$$\begin{array}{l} \Theta_{t}^{G}=\frac{1}{M}\overset{M}{\underset{m=1}{\sum }}\Theta_{bt}^{G_{m}},\;\Theta_{t}^{D}=\frac{1}{M}\overset{M}{\underset{m=1}{\sum }}\Theta_{bt}^{D_{m}} \end{array}$$ (31)

This aggregation process allowed the global model to improve without requiring the central server to access the actual EEG data. After aggregation, the updated global models were sent back to the clients, and the process was repeated over multiple global epochs T. The entire process is repeated until the model converges or until a predefined number of global epochs are completed.

#### 2.2.8 Differential privacy with Rényi differential privacy (RDP)

To provide a formal privacy guarantee for the real EEG data used during discriminator training, we employ Differentially Private Stochastic Gradient Descent (DP-SGD) together with Rényi Differential Privacy (RDP) accounting. The DP-SGD mechanism consists of per-example gradient clipping followed by the addition of Gaussian noise to the aggregated discriminator gradient, as defined in Equations 26–28.

Let α denote a Rényi order, and let A denote a finite set of Rényi orders used for accounting. For a subsampled Gaussian mechanism with sampling probability *q* and DP noise multiplier σ_DP_, let


$$\begin{array}{l} R_{\alpha }=R_{\alpha }\left(q,\sigma_{\text{DP}}\right) \end{array}$$ (32)

denote the RDP cost of one DP-SGD step at order α as defined in Equation 32. After *S* discriminator updates, RDP composes additively according to Equation 33:


$$\begin{array}{l} \epsilon_{\text{RDP}}^{\left(\alpha \right)}=SR_{\alpha }\left(q,\sigma_{\text{DP}}\right) \end{array}$$ (33)

The resulting RDP guarantee is converted to an (ϵ, δ)-differential privacy guarantee by minimizing over the finite set of Rényi orders:


$$\begin{array}{l} \epsilon =\underset{\alpha \in A}{\min}\left[SR_{\alpha }\left(q,\sigma_{\text{DP}}\right)+\frac{\log\left(1/\delta \right)}{\alpha -1}\right] \end{array}$$ (34)

where *q* is the sampling probability of an individual DP-SGD step, σ_DP_ is the DP noise multiplier, *S* is the total number of discriminator DP-SGD updates affecting the considered client data, and δ is the target probability parameter. In the present experiments, the clipping norm is *C* = 1.0 and δ = 10^−3^. The sampling probability and total number of updates are determined from the actual mini-batch training procedure rather than being inferred solely from the number of local epochs.

For client *m*, when fixed-size mini-batches of size B are used with drop_last = True, the number of complete local batches per epoch is calculated using Equation 35:


$$\begin{array}{l} S_{\text{epoch}}^{\left(m\right)}=\left\lfloor \frac{N_{m}}{B}\right\rfloor \end{array}$$ (35)

and, for T communication rounds and L local epochs, the total number of discriminator DP-SGD updates for client *m* is given by Equation 36:


$$\begin{array}{l} S_{m}=TLS_{\text{epoch}}^{\left(m\right)} \end{array}$$ (36)

The privacy guarantee for the federated experiment is reported conservatively using the largest privacy loss among the participating clients, as formulated in Equation 37:


$$\begin{array}{l} \epsilon_{\text{Fed}}=\underset{m\in \left\{1,\ldots ,M\right\}}{\max}\epsilon_{m} \end{array}$$ (37)

The number of clients M is therefore not multiplied directly into the privacy loss. Each client's privacy loss is accumulated over the DP-SGD steps applied to that client's data. When client participation is subsampled, the corresponding client-participation probability must additionally be incorporated into the privacy accountant. In the present experiments, all four simulated clients participate in each communication round.

The temporally correlated noise defined in Equation 20 is not included as an additional independent Gaussian mechanism in Equation 34. This is because its autoregressive construction introduces temporal dependence between successive perturbations. Its influence is therefore evaluated separately as part of the generator's temporal stochasticity and through the experimental privacy–utility analysis. The formal (ϵ, δ) guarantee reported in this work is based on the DP-SGD mechanism and its RDP composition.

This distinction separates the temporal perturbation used to improve the stochastic temporal behavior of the SNN generator from the Gaussian perturbation applied to clipped discriminator gradients for formal differential privacy.

### 2.3 Experimental setup

#### 2.3.1 Dataset

We used the BCI IV 2A dataset, which contains EEG recordings of nine subjects identified as A1 through A9, performing four motor imagery tasks (MIT) (Brunner et al., 2008). The data of each subject are divided into two sessions: Session I and Session II. For Dataset 2A, both Session I and Session II are characterized by the same data shape (including artifacts), denoted (E_ld_, Trial_N_, D_tp_). In this notation, E_ld_ represents the number of electrodes or EEG channels, which is 22. The signals are filtered using a bandpass filter (b_pf_) between 0.5 and 100 Hz, and a notch filter (n_f_) is applied at 50 Hz. Trial_N_ signifies the total number of trials per session, which is 2,592, including artifacts. D_tp_ refers to the number of data points per trial, calculated as D_tp_ = time_s_×s_f_, where s_f_ is the sampling rate. Given that the MIT lasts for 4 s with a s_f_ of 250 Hz, D_tp_ is 4 × 250 = 1, 000. Therefore, for each session (containing nine subjects), the shape of the dataset, including artifacts, is (22, 2,592, 1,000), where for each subject's data, the shape is (22, 288, 1,000).

We used C3, C4, and Cz EEG channels in our study instead of 22 EEG channels as these channels are effective in capturing brain patterns associated with the recognition of imaginary movement states (Hu et al., 2014). We focused on the C3, Cz, and C4 channels because these electrodes are located over the sensorimotor cortex and are particularly relevant to motor-imagery activity (Hu et al., 2014). This reduced-channel configuration has also been adopted in previous studies using the BCI Competition IV Dataset 2a, including Jia et al. (2020), who used three channels (C3, Cz, and C4) in MMCNN, and Wu et al. (2025), who similarly selected these three channels from the original 22-channels.

We removed artifacts from both sessions, and used Session I as train data (Tr), and Session II as test data (Te). Therefore, each subject had a variable shape of input data for train and test sets as shown below:

- Subject A1: Tr – (273, 3, 1, 000), labels – (273, 1), Te – (281, 3, 1, 000), labels – (281, 1)
- Subject A2: Tr – (270, 3, 1, 000), labels – (270, 1), Te – (283, 3, 1, 000), labels – (283, 1)
- Subject A3: Tr – (270, 3, 1, 000), labels – (270, 1), Te – (273, 3, 1, 000), labels – (273, 1)
- Subject A4: Tr – (262, 3, 1, 000), labels – (262, 1), Te – (228, 3, 1, 000), labels – (228, 1)
- Subject A5: Tr – (262, 3, 1, 000), labels – (262, 1), Te – (276, 3, 1, 000), labels – (276, 1)
- Subject A6: Tr – (219, 3, 1, 000), labels – (219, 1), Te – (221, 3, 1, 000), labels – (221, 1)
- Subject A7: Tr – (271, 3, 1, 000), labels – (271, 1), Te – (277, 3, 1, 000), labels – (277, 1)
- Subject A8: Tr – (264, 3, 1, 000), labels – (264, 1), Te – (271, 3, 1, 000), labels – (271, 1)
- Subject A9: Tr – (237, 3, 1, 000), labels – (237, 1), Te – (264, 3, 1, 000), labels – (264, 1)

The labels in the dataset correspond to four motor imagery tasks. These tasks are classified as follows (Brunner et al., 2008): Class 1 (left), represented by event type 769 (0 × 0301) and referred to as CL1; Class 2 (right), represented by event type 770 (0 × 0302) and referred to as CL2; Class 3 (foot), represented by event type 771 (0 × 0303) and referred to as CL3; and Class 4 (tongue), represented by event type 772 (0 × 0304) and referred to as CL4. For generating synthetic data, only the training data from Session I was used.

#### 2.3.2 Data pre-processing

The EEG data for each channel is normalized to the range [0, 1] using the min-max normalization method. The normalized value, *E*_*i*_, is calculated as follows in Equation 38:


$$\begin{array}{l} E_{i}=\frac{X_{i}-X_{\min}}{X_{\max}-X_{\min}} \end{array}$$ (38)

where *X*_*i*_ represents the data point from channel *i*, and *X*_min_ and *X*_max_ are the minimum and maximum values of the EEG data for channel *i*, respectively.

#### 2.3.3 Model architecture

The Spiking GAN architecture consists of a Spiking generator and an ANN-based discriminator. Using temporal patterns observed in spiking neurons, the Spiking Generator generates synthetic EEG data. It preserves important temporal properties that resemble biological cerebral activity, while it converts a low-dimensional latent space, a 100-dimensional vector, into high-dimensional EEG signals. Starting from a typical normal distribution, this latent vector is then concatenated with a label embedding to guarantee that the generator outputs EEG data specific to a class. By adding pertinent information to the latent space, this combination conditions the generating process on the intended label.

Three fully connected layers make up the architecture of the generator. To begin the transformation to a high-dimensional EEG representation, the first layer processes the concatenated latent vector and the embedding of the label, thus increasing the dimensionality of the input. The second layer continues this process by increasing feature complexity. The third layer, which peaks at 1,000 neurons, fully contains the features required to generate the EEG signal. Leaky Integrate-and-Fire (LIF) neurons generate spiking behavior after each fully connected layer, simulating membrane potential dynamics, and ensuring that the resulting data resemble actual EEG signals. The last layer uses a Sigmoid activation function to scale the output to the range [0, 1], which corresponds to the normalized EEG data format. It has 3,000 neurons, or three channels with 1,000 datapoints each.

The generated EEG data, authenticated by the ANN-based discriminator, is gradually reduced in dimensionality while extracting important features that distinguish real from fake data. In the first fully connected layer, it starts with the entire 3,000-dimensional EEG vector, concatenates it with the label embedding, and reduces it to 1,000 neurons. Subsequent layers of this downsampling technique focus on fine-tuning features indicative of data authenticity, with the second layer reducing the dimensionality to 750 neurons and the third to 500 neurons. Before the final layer restores the dimensionality to 1,000 neurons, the fourth layer significantly reduces it to 750 neurons, enabling wider exploration of the feature space. Before the final categorization, this symmetric structure guarantees a rich representation of the input data. The output layer, which consists of a single neuron, uses a Sigmoid activation function to output a value between 0 and 1. This value indicates the chance that the input data is real and signals the generator to produce more realistic synthetic data. During training, the discriminator is optimized using DP-SGD. A clipping norm of *C* = 1.0 is applied to each per-example discriminator gradient, followed by Gaussian perturbation controlled by the DP noise multiplier σ_DP_. The mini-batch size is B=8, with drop_last = True, and the resulting number of complete mini-batches is determined separately for each client according to its artifact-free local dataset size.

## 3 Results and discussion

We modified several existing GAN-based models to align their training procedures with the Rényi Differential Privacy (RDP) framework, enabling a privacy-preserving comparison with the proposed federated Spiking GAN. Among these models were TimeGAN (Yoon et al., 2019), RCGAN (Esteban et al., 2017), CRNN-GAN (Mogren, 2016), and Clare-GAN (Arnout et al., 2021). By incorporating per-example gradient clipping and Gaussian perturbation into the discriminator training of their respective architectures, we developed RDP-TimeGAN, RDP-RCGAN, RDP-CRNN-GAN, and RDP-Clare-GAN. A clipping norm of 1.0 was employed. The total number of iterations for these methods is set as 200. A finite set of RDP orders is used as A. The other parameter details are shown in Table 1. Importantly, the noise multiplier is a parameter of the DP mechanism rather than the privacy budget itself. For each method, the corresponding (ϵ, δ) privacy guarantee is calculated using the corrected RDP accountant based on the actual sampling rate and number of DP-SGD updates. Thus, methods may have different ϵ values at the same noise multiplier when their training schedules differ. This provides a more rigorous privacy–utility comparison between the proposed federated Spiking GAN and the non-federated RDP baselines.

**Table 1:** Common parameters and variables used in the experiments.

Parameter	Value
Generator input	100-dimensional latent vector + class label embedding
Generator hidden layers	Three fully connected layers with LIF neurons
Generator output	3,000 neurons (three channels × 1,000 datapoints)
Discriminator input	3,000-dimensional EEG vector + class label embedding
Privacy budget (ϵ)	[1, 2, 3, 4, 5, 6, 7, 8, 9, 10]
Number of communication rounds (T)	10 communication rounds
Number of local epochs (L)	4
Number of clients(ℳ)	4
Privacy parameter (δ)	10^−3^
Clipping norm (*C*)	1.0
Learning rate (η)	0.0002
drop_last	True
Data split	Equally among clients

The federated experiments were conducted independently for each subject, with each subject's EEG trials partitioned among four clients. Data from different subjects were not pooled within the same federated experiment. This subject-specific setting was used to evaluate privacy-preserving synthetic EEG generation while maintaining subject-level separation. However, heterogeneous non-IID client distributions were not explicitly evaluated and remain an important direction for future work. Since the data-dependent contribution score *c*_*m*_ is not separately privatized, its privacy cost is not included in the reported DP-SGD/RDP accounting. Future work will focus on privatizing *c*_*m*_ and composing its privacy cost with the DP-SGD/RDP mechanism.

### 3.1 Evaluation scenarios

We performed the following tests to evaluate the usability of the generated EEG data using two well-known classification architectures: EEGNet (Lawhern et al., 2018), which generalizes across the BCI paradigms, and ATCNet (Altaheri et al., 2022), which shows excellent performance with motor imagery Dataset 2A.

- ***Train*_(*Tr*+*Sy*)_, *Test*_(*Te*)_:** Trained on real EEG samples of the train set plus the synthetic dataset (augmented dataset), and tested on real EEG samples of the test dataset.
- ***Train*_(*Tr*_50_+*Sy*_50_)_, *Test*_(*Te*)_:** Trained on 50% real EEG samples from the train set plus the 50% synthetic dataset, and tested on real EEG samples of the test dataset.
- ***Train*_(*Tr*)_, *Test*_(*Sy*)_:** Trained only on the real EEG samples of the train dataset, and tested on the synthetic dataset.
- ***Train*_(*Sy*)_, *Test*_(*Tr*)_:** Trained on a synthetic dataset, and tested on real EEG samples of the training dataset.
- ***Train*_(*Sy*)_, *Test*_(*Te*)_:** Trained on a synthetic dataset, and tested on real EEG samples of the test dataset.

We evaluated the classification performance of each model over 30 runs to ensure the validity of our findings. The real test set (*Te*) and the real training set (*Tr*) for each subject were denoted by the A0PE and A0PT datasets, respectively, with “P” representing the subject identifier. We created synthetic data using the entire A0PT dataset for our proposed method and the comparison approaches. Following the creation of the synthetic data, we trained the EEGNet and ATCNet classifiers and analyzed their performance across a variety of scenarios. We conducted experiments with various temporal correlation coefficients (ς) ranging from 0.1 to 0.9. Our observations indicated that data quality, measured in terms of accuracy for the five different evaluation scenarios, from *Train*_(*Tr*+*Sy*)_, *Test*_(*Te*)_ to *Train*_(*Sy*)_, *Test*_(*Te*)_, was optimal at a ς value of 0.55. Consequently, we used this value of ς for all subsequent comparisons with other methods.

The experimental results for our proposed Spiking GAN model, RDP-TimeGAN, RDP-RCGAN, RDP-CRNN-GAN, and RDP-Clare-GAN are shown in Figures 3–7. These figures show the comparison of four state-of-the-art models evaluated using ATCNet and EEGNet for subject A1. These figures illustrate the models' performance across various noise multipliers for one subject (A1) as an example representation. The results of the remaining subjects (A2-A9) have been included in the Supplementary materials. These also illustrate how different levels of noise impact the models' ability to preserve privacy while maintaining efficient data-generation capabilities. These evaluations highlight the crucial trade-off between privacy (quantified by ϵ) and model accuracy. We observed that both architectures give comparable performance for most of the subjects but with a high degree of variability highlighting the need for individualized utility optimization (deep learning models) for similar privacy budgets.

**Figure 3:**
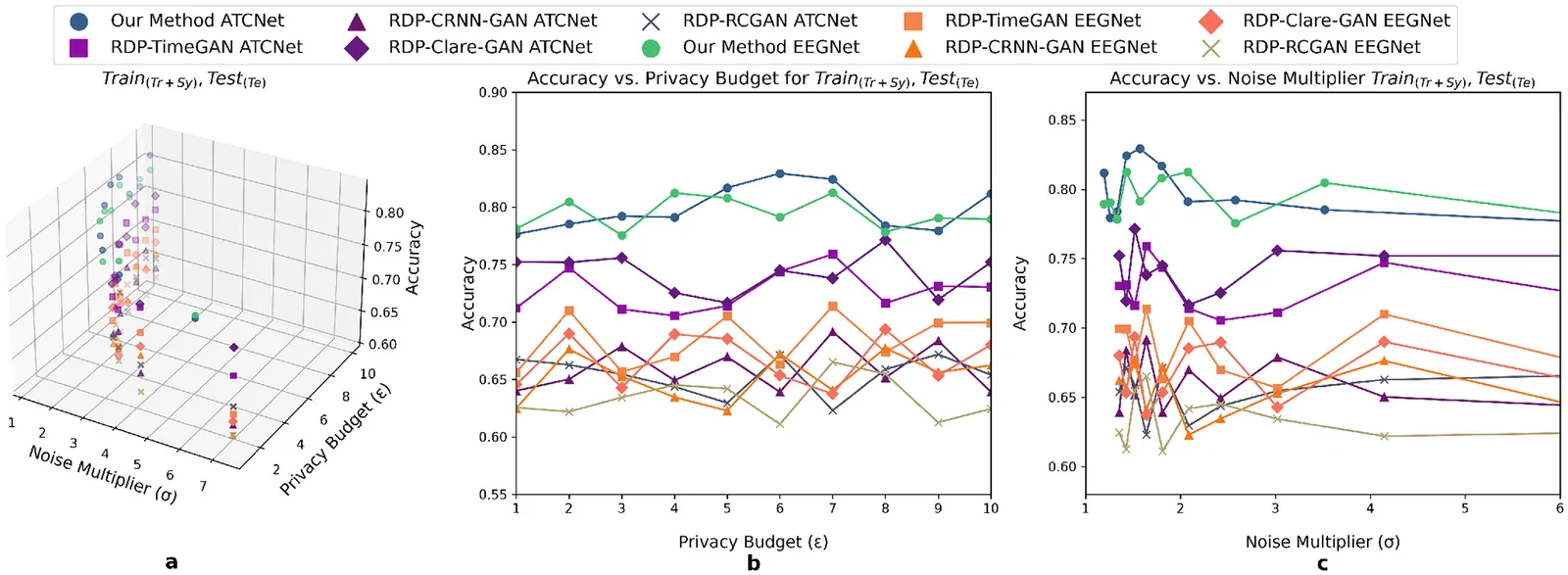
Comparison of different models under various privacy budgets and noise multipliers for subject A1 [(*Train*_(*Tr*+*Sy*)_, *Test*_(*Te*)_]. The simultaneous impact of the privacy budget and noise multiplier is shown via a 3D surface plot in panel **(a)** with individual parameter sensitivities isolated in panels **(b)** and **(c)**.

**Figure 4:**
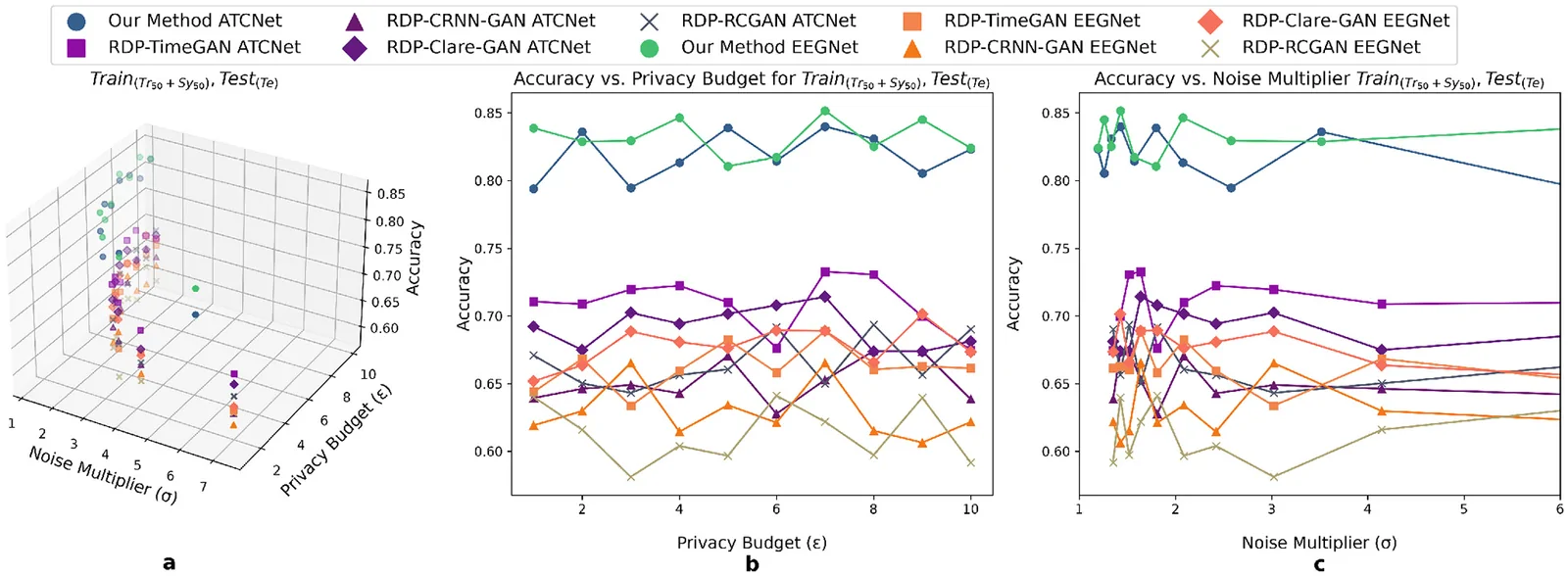
Comparison of different models under various privacy budgets and noise multipliers for subject A1 [*Train*_(*Tr*_50_+*Sy*_50_)_, *Test*_(*Te*)_]. The simultaneous impact of the privacy budget and noise multiplier is shown via a 3D surface plot in panel **(a)** with individual parameter sensitivities isolated in panels **(b)** and **(c)**.

**Figure 5:**
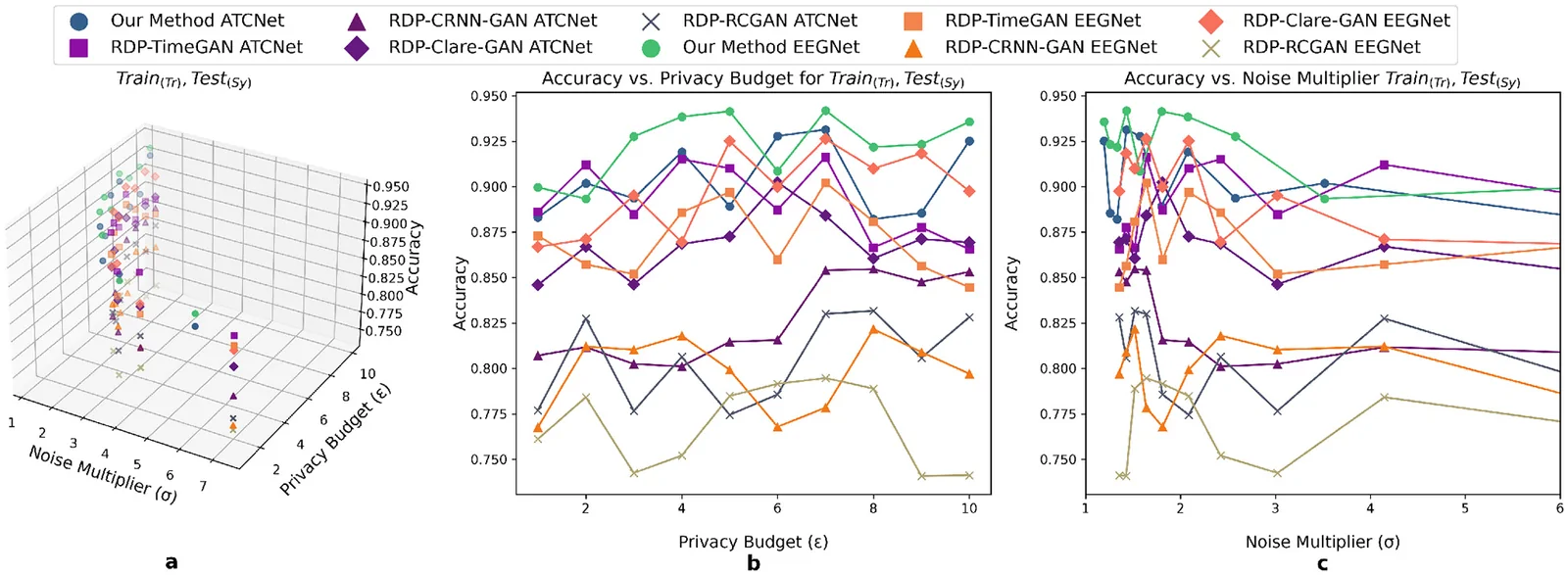
Comparison of different models under various privacy budgets and noise multipliers for subject A1 [*Train*_(*Tr*)_, *Test*_(*Sy*)_]. The simultaneous impact of the privacy budget and noise multiplier is shown via a 3D surface plot in panel **(a)** with individual parameter sensitivities isolated in panels **(b)** and **(c)**.

**Figure 6:**
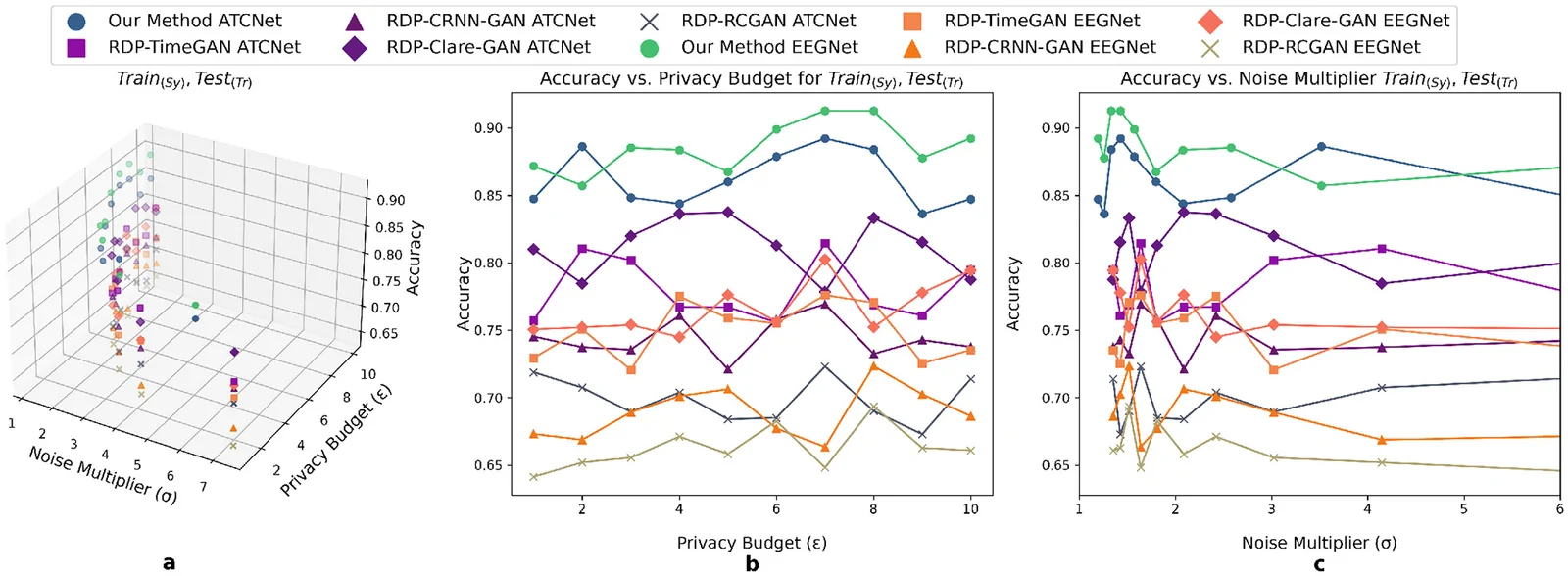
Comparison of different models under various privacy budgets and noise multipliers for subject A1 [*Train*_(*Sy*)_, *Test*_(*Tr*)_]. The simultaneous impact of the privacy budget and noise multiplier is shown via a 3D surface plot in panel **(a)** with individual parameter sensitivities isolated in panels **(b)** and **(c)**.

**Figure 7:**
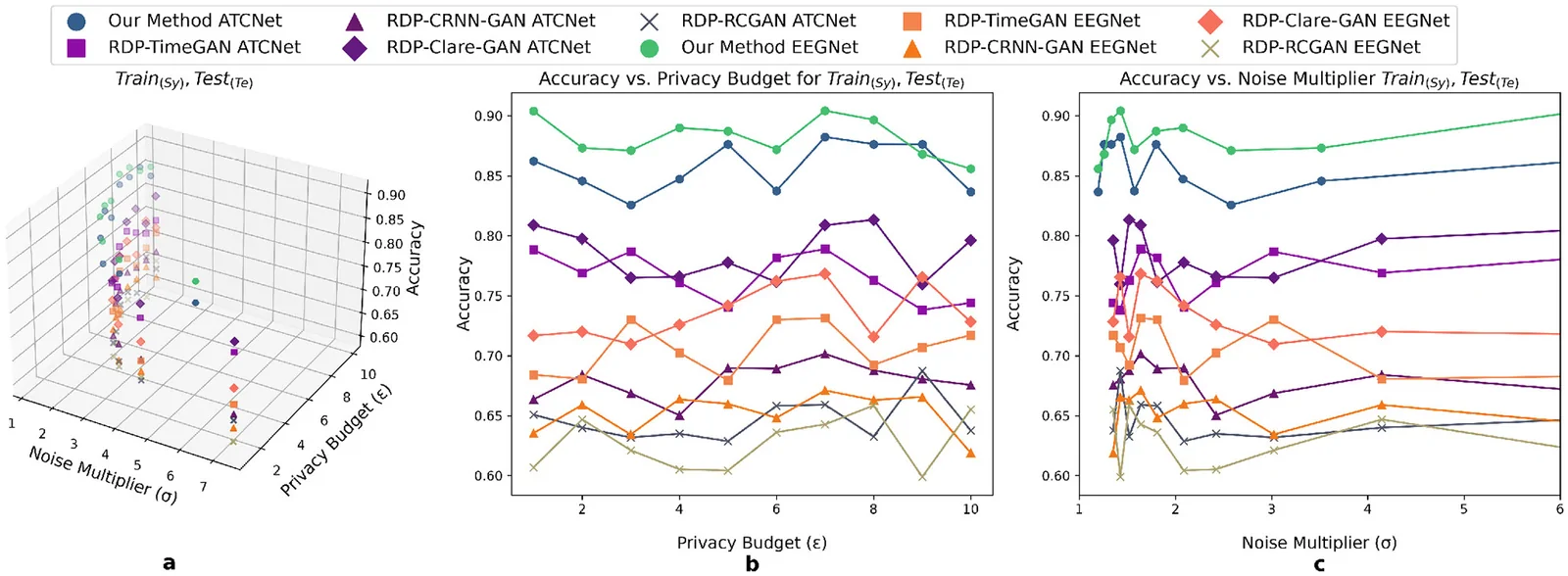
Comparison of different models under various privacy budgets and noise multipliers for subject A1 [*Train*_(*Sy*)_, *Test*_(*Te*)_]. The simultaneous impact of the privacy budget and noise multiplier is shown via a 3D surface plot in panel **(a)** with individual parameter sensitivities isolated in panels **(b)** and **(c)**.

We also found that for our method, the accuracy of both classifiers (EEGNet and ATCNet) for scenario 1, *Train*_(*Tr*+*Sy*)_, *Test*_(*Te*)_ was comparable to or slightly less than that for scenario 2, *Train*_(*Tr*_50_+*Sy*_50_)_, *Test*_(*Te*)_. This suggests that reducing the real training data by 50% and augmenting with 50% synthetic data maintains or even slightly improves accuracy. This indicates that our privacy-preserving model can be deployed in BCI environments which are less sensitive to accuracy variability, such as BCI games. In contrast, other models such as RDP-TimeGAN, RDP-RCGAN, RDP-CRNN-GAN, and RDP-Clare-GAN showed a decrease in accuracy under similar conditions. The difference in accuracy between *Train*_(*Tr*)_, *Test*_(*Sy*)_ and *Train*_(*Sy*)_, *Test*_(*Tr*)_ is more noticeable compared to other methods. However, in our approach, this difference is smaller, indicating the high quality of the synthetic EEG data generated. Furthermore, in the case of *Train*_(*Sy*)_, *Test*_(*Te*)_, our method produces better accuracy than other approaches, suggesting a better data utility.

Figure 3 compares the classification accuracy obtained from EEG signals generated by the proposed method and the RDP-based baseline methods under different privacy budgets and noise multipliers. As shown in Figure 3b, the proposed method achieves the highest accuracy for ATCNet across most privacy-budget settings, reaching approximately 0.83, while the proposed EEGNet also maintains consistently high accuracy of approximately 0.78–0.81. This indicates that the proposed framework preserves the discriminative temporal characteristics of EEG signals under different privacy levels. Among the baseline methods, RDP-TimeGAN generally performs best, with accuracy around 0.76 for ATCNet and 0.75 for EEGNet. However, it remains below the proposed method in most settings. RDP-CLARE-GAN shows intermediate performance, whereas RDP-CRNN-GAN and RDP-RCGAN generally produce lower and more variable accuracies. The lower performance of these baseline methods may stem from differences in their generative architectures and their ability to preserve the temporal and discriminative characteristics of EEG signals while introducing differential privacy noise. Figure 3c further shows that the proposed method maintains relatively stable accuracy across increasing noise multipliers, particularly for ATCNet, whereas the baseline methods exhibit larger fluctuations and generally lower accuracy. Overall, these results suggest that the combination of the proposed spiking representation, federated training, and privacy-preserving mechanism provides a more robust balance between EEG utility and privacy than the evaluated RDP-based baseline methods.

### 3.2 Data fidelity

The fidelity and usability of the generated EEG data is evaluated using Dynamic Time Warping (DTW) (Muller, 2007; Li, 2021) and Spectral Similarity (SS) (Gupta et al., 2005) techniques. DTW aligns the sequences to minimize the overall distance in order to determine how similar the actual and synthetic EEG signals are to each other. DTW is useful for assessing the temporal dynamics of the data. Low values indicate that the temporal dynamics of the synthetic data closely follows the real data. SS gives a comparison of the power spectral density (PSD) between synthetic and actual EEG data. A high value of spectral similarity means that the synthetic data maintain the frequency characteristics of the genuine data. The SS score ranges from 0 to 1. The range of DTW and SS metrics obtained for the generated EEG data, shown in Figures 8, 9, demonstrates that our method produces high-fidelity synthetic data that closely resemble the real data (Geng and Chen, 2021). The inter-subject variability observed in the DTW and SS scores suggests that the synthetic data may not have fully integrated the individual-specific characteristics of the EEG signals. This variability could potentially be reduced by using more subject-specific modeling approaches in the synthetic data generation process. Overall, the fidelity of the synthetic data is high, despite considerable variation between subjects. This implies that our technique preserves the essential features of EEG signals, such as frequency content and temporal dynamics, while adapting well to different subjects. DTW and SS were used to assess the temporal and spectral characteristics preserved in the synthetic EEG generated by our method. These results demonstrate signal fidelity sufficient for downstream tasks, as shown by the accuracy of generated synthetic data (Figures 3–7), while preserving privacy, but do not establish superior fidelity relative to other models.

**Figure 8:**
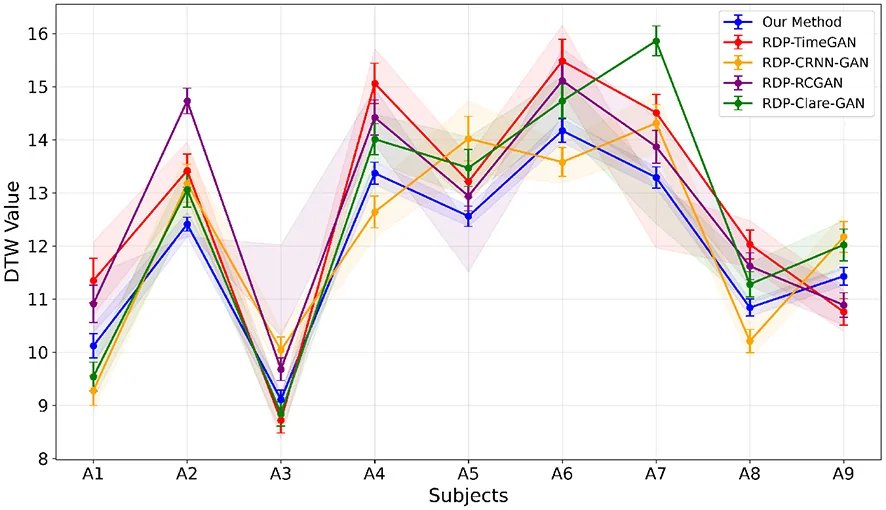
The plots shows the mean dynamic time warping (DTW) values with standard deviations and ranges obtained for the generated EEG data of all subjects, A1–A9.

**Figure 9:**
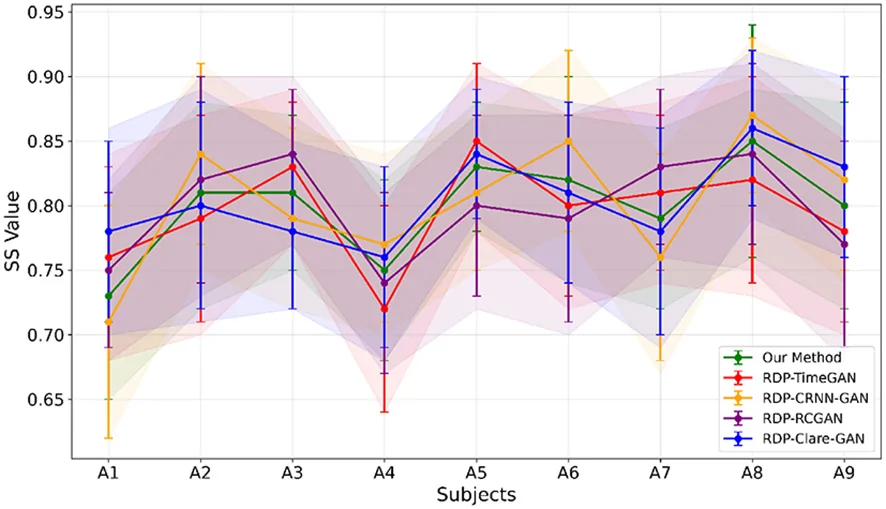
The plot shows the mean spectral similarity score (SS) values with standard deviations ranges obtained for the generated EEG data of all subjects, A1–A9.

### 3.3 Visualization of high-dimensional EEG data using 3D t-SNE

In order to compare the original with generated EEG data, we used t-Distributed Stochastic Neighbor Embedding (t-SNE), which visualizes high-dimensional datasets by projecting from higher dimensions to lower ones (Van der Maaten and Hinton, 2008). Through the t-SNE visualizations, the degree of convergence of the synthetic data generation process can be assessed, facilitating the identification of similarities or dissimilarities between the real and synthetic datasets.

Figure 10A illustrates the spatial distribution of the real and synthetic EEG data points generated by our proposed Spiking GAN model and Figures 10B–E of four other models including RDP-TimeGAN, RDP-RCGAN, RDP-CRNN-GAN and RDP-Clare-GAN. Due to the large number of images and their file size, only one subject's results are shown as a representation of the dataset.

**Figure 10:**
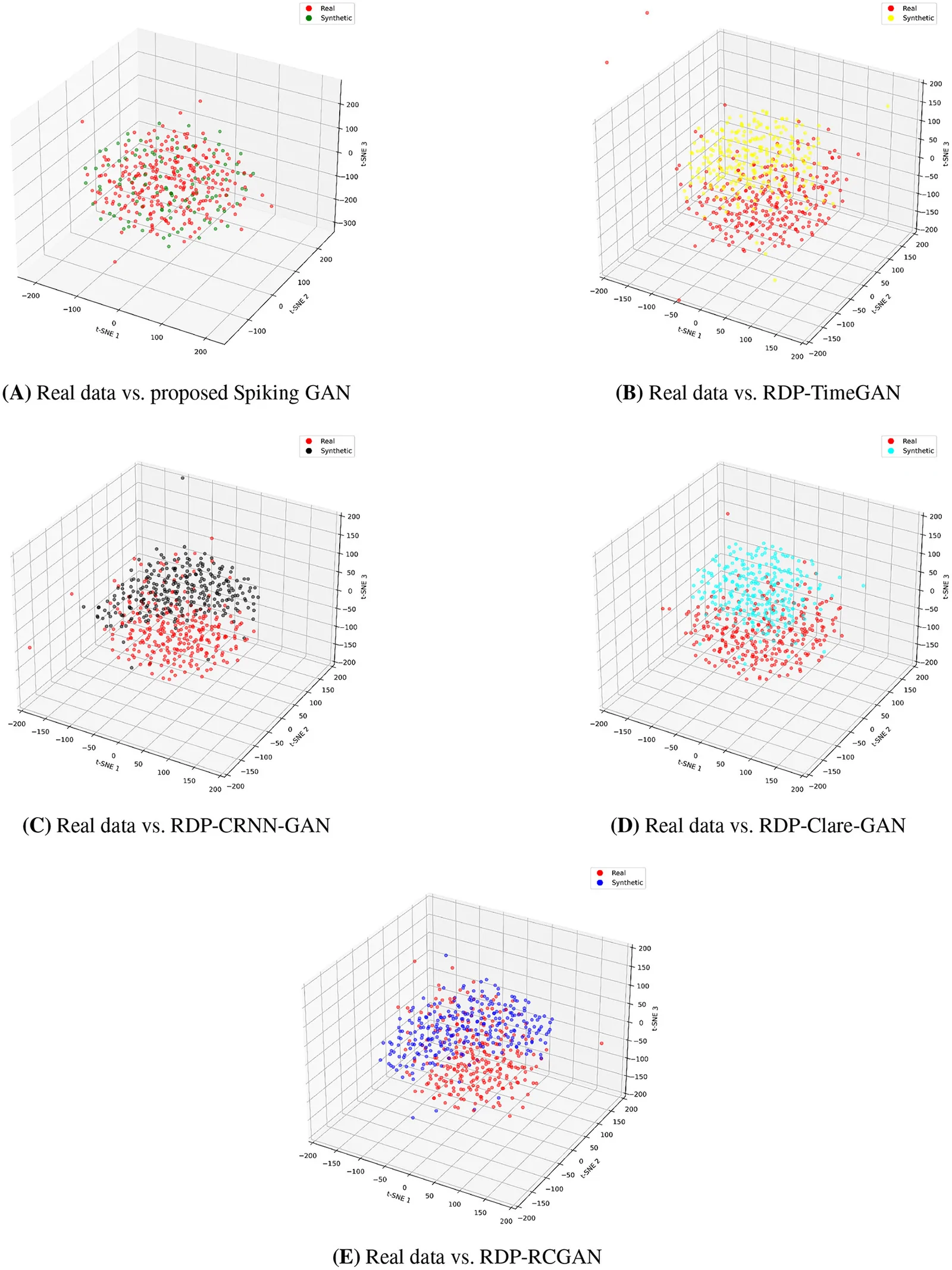
Three-dimensional t-SNE visualization of high-dimensional EEG data for subject A1, comparing the distribution of real data (red color) with synthetic data generated by different models. Panels **(A–E)** present a side-by-side distribution comparison of the real data baseline against synthetic data generated by the proposed Spiking GAN (A) and the respective comparative frameworks across panels **(B–E)**.

The clusters and patterns observed in the t-SNE plots demonstrate that our Spiking GAN model generates synthetic EEG data that closely approximate the distribution of authentic EEG data, ensuring privacy preservation while maintaining high data fidelity. The performance is also considerably better than that of the RDP-CRNN-GAN and RDP-RCGAN models, which exhibit a greater degree of homogeneity relative to those of RDP-TimeGAN and RDP-Clare-GAN.

Experimental results consistently indicate that our proposed Spiking GAN model strikes a balance between privacy and accuracy more effectively than the baseline models. Furthermore, the lower epsilon values achieved signify enhanced privacy preservation while maintaining high data fidelity and usability.

### 3.4 Full black-box attack

To thoroughly evaluate the privacy guarantees of our synthetic EEG data generation model, we performed a Full Black-Box Attack (FBA) similar to Gwon et al. (2024). Here, an adversary has very restricted access, solely to the synthetic data generated by the model.

The objective of the adversary is to infer whether a specific real data point *X* was used during the training of the model by analyzing synthetic data.


$$R=\arg\underset{\hat{X}}{\min}L\left(X,\hat{X}\right)$$

where, Euclidean distance, *L* is the metric used to calculate the difference between the generated data point $\hat{X}$ and the real data point *X* and R denotes the reconstructed data point, which is the data point of the generated set that is closest to the target data point. The adversary is informed of a potential privacy violation when the calculated distance *L*(*X, R*) is found to be less than a preset threshold *T*_κ_. This is because it raises the possibility that the original data point *X* was included in the training dataset. The following is a mathematical expression for the attack:


$$A\left(X\right)=\{\begin{array}{ll} 1 & \text{ if }L\left(X,R\right)\leq T_{\kappa }\\ 0 & \text{otherwise} \end{array}$$

where, *A*(*X*) is a binary function that returns 1 if the distance is within the threshold *T*_κ_, indicating a successful inference, and 0 otherwise.

#### 3.4.1 Full black-box attack results

The number of samples leaked during the Full Black-box Attack is measured as a part of the total dataset based on the output of the evaluation function, A(X). The results of the attack are summarized in Figure 11. Due to the large number of images and their file size, only one subject's results are shown as a representation of the dataset. Additional figures for other subjects are included in the Supplementary material. One important finding from the data is that our model shows great resilience against the FBA with few successful inferences when the ϵ is kept low. In line with the anticipated trade-off between privacy and utility in differential privacy frameworks, the model becomes more vulnerable to inference attacks as ϵ increases.

**Figure 11:**
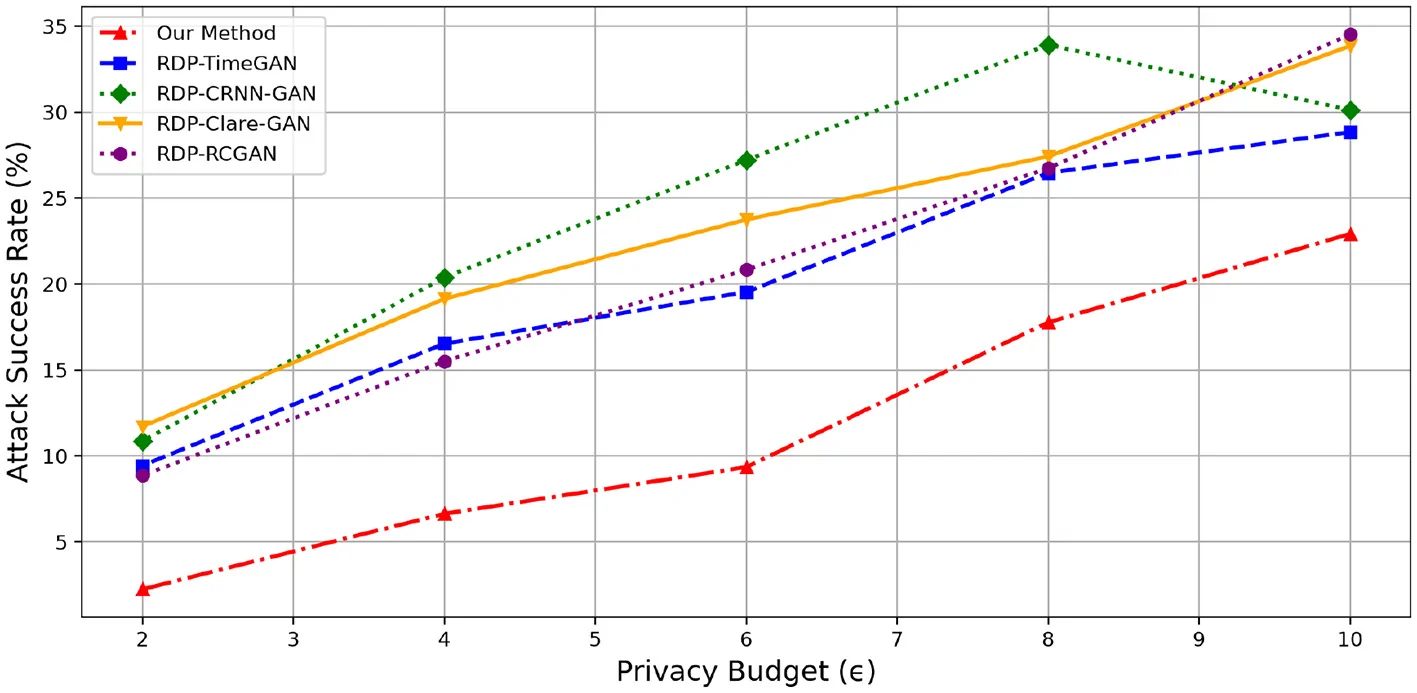
A comparison of attack success rate (%) for subject A1 across different ϵ values and privacy models. A higher percentage signifies a more successful attack, whereas a lower percentage indicates better privacy protection.

For this evaluation, we set *T*_κ_ at 0.05, also observed in Gwon et al. (2024), which balances the trade-off between the sensitivity of the attack and the practical utility of the generated data. Similarly, our findings indicate that to achieve the balance between privacy and utility of the model, ϵ should be maintained within a range based on the application requirement. Too low ϵ values increase privacy but reduce utility, and too high values increase susceptibility to attacks, as shown in Figure 11.

### 3.5 Ablation study

To investigate the contribution of the principal components of the proposed framework, an ablation study was conducted by selectively removing or replacing individual components of the proposed method. The study considers the SNN-based generator, federated training, and the privacy-preserving mechanism as separate components. The objective is to determine whether the observed performance is attributable to the complete combination of these components or can be obtained by a conventional generative architecture alone.

Four additional experimental conditions were considered. First, the SNN-based generator was replaced with a conventional CNN-based generator while retaining the remaining experimental conditions. The condition without the SNN generator is denoted as *WOSNN*. This condition evaluates the contribution of spiking temporal dynamics. Second, the federated training procedure was replaced by conventional non-federated GAN training, allowing the contribution of the federated learning framework to be examined. The condition without federated learning is denoted as *WOFL*. Third, the temporally correlated noise and the RDP mechanism were removed, resulting in a non-private GAN condition. The condition without the correlated noise and formal privacy mechanism is denoted as *WOCRN*. This condition provides a reference for evaluating the utility cost associated with privacy-preserving training. Finally, an EEGNet classifier trained only on the original real training data and evaluated on the original real test data, denoted as the *Real-only* baseline, was included to provide a reference without synthetic-data augmentation.

For the first three ablation conditions, the generated EEG samples were used together with the real training data for augmentation, and the resulting classifiers were evaluated on the real test data. We used EEGNet for all ablation conditions. The privacy-preserving ablation conditions were evaluated at a common privacy budget of ϵ = 4, whereas the real-data-only classifier does not have an associated synthetic-data privacy budget because it does not use generated data. The non-private GAN condition is similarly reported as a non-private reference and is therefore not assigned a finite DP privacy budget.

The ablation results in Figure 12 show a clear privacy–utility trade-off. The WOCRN condition achieves higher classification accuracy than the privacy-preserving conditions because removing the privacy mechanism eliminates the utility cost associated with RDP and allows the GAN to generate less privacy-constrained synthetic samples. Its accuracy is also higher than the Real-only baseline, indicating that, in this experiment, augmenting the real training data with non-private synthetic EEG samples provides additional useful training information.

**Figure 12:**
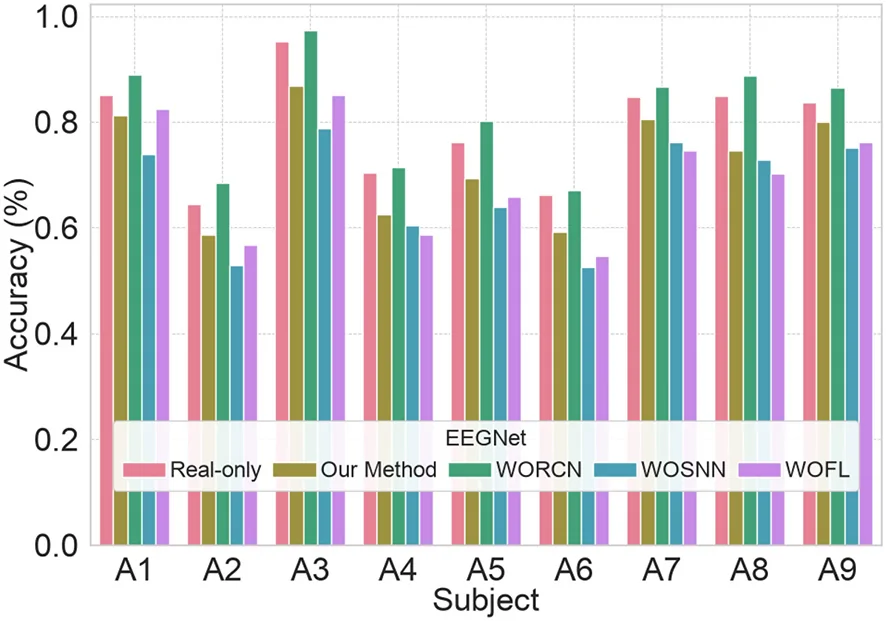
Ablation study for all subjects. *WOSNN* represents the condition in which there is no SNN generator. *WORCN* represents the condition in which there is no correlated noise and RDP. *WOFL* represents the condition in which there is no federated learning. *Real* − *only* represents the condition in which it is trained only on the original real training data and evaluated on the original real test data. The results are shown here for *OurMethod*, *WOSNN*, *WOFL* while ϵ is 4.

In contrast, the privacy-preserving conditions generally achieve lower accuracy than the non-private WOCRN condition because the generated samples are produced under privacy constraints. Nevertheless, the proposed SNN-based federated method achieves better utility than the WOSNN condition, indicating that the SNN generator provides a beneficial contribution beyond replacing it with a conventional CNN generator. Similarly, the comparison with WOFL shows the effect of federated training and indicates that retaining the federated learning framework provides additional benefit under the evaluated experimental setting. Overall, the ablation results suggest that the performance of the proposed method is not attributable to a single component; rather, the SNN generator, federated training, and privacy-preserving mechanism jointly determine the observed privacy–utility trade-off.

## 4 Conclusion

In this study, we introduce a novel methodology for generating privacy-preserving synthetic EEG data through the application of a Federated Spiking GAN framework, which incorporates spiking neural networks (SNNs) in the generator, an artificial neural network (ANN)-based discriminator, and a temporally correlated noise model. Unlike other methods, ours takes advantage of the temporal dynamics inherent in SNNs to effectively capture the essential characteristics of EEG signals. By integrating Rényi Differential Privacy (RDP) into the federated learning paradigm, we ensure robust privacy guarantees without compromising data utility. Our experiments demonstrate the efficacy of our approach in producing high-fidelity synthetic EEG data while maintaining a balance between privacy and utility. Extensive comparative analysis with other GAN-based privacy-preserving methodologies, such as RDP-TimeGAN, RDP-CRNN-GAN, RDP-RCGAN, and RDP-Clare-GAN, underscores the performance of our method in preserving the temporal dynamics of EEG signals and providing strong privacy.

Our methodology advances current practices in generating privacy-preserving synthetic data and paves the way for emerging applications that require both data fidelity and privacy across various clinical and research contexts. Future research will investigate additional privacy-preserving techniques, including integration with secure multi-party computation, to further enhance privacy guarantees. Furthermore, we intend to extend our approach to other types of neural data, such as fMRI and MEG, and explore integrating multimodal data to enable more comprehensive neural data synthesis.

## Data Availability

The dataset used in this study is publicly available and can be downloaded from https://bbci.de/competition/iv/download/.
