# Supplementary material for: Federated Spiking GANs with Rényi Differential Privacy for privacy-preserving synthetic EEG generation in brain–computer interfaces
Source: Front Hum Neurosci. 2026 Oct 14;20:1909432. doi: 10.3389/fnhum.2026.1909432 (PMC13713703; doi:10.3389/fnhum.2026.1909432)
Supplement: Supplementary file 1 [file Data_Sheet_1.pdf]

Supplementary Material

1 EVALUATION SCENARIOS:

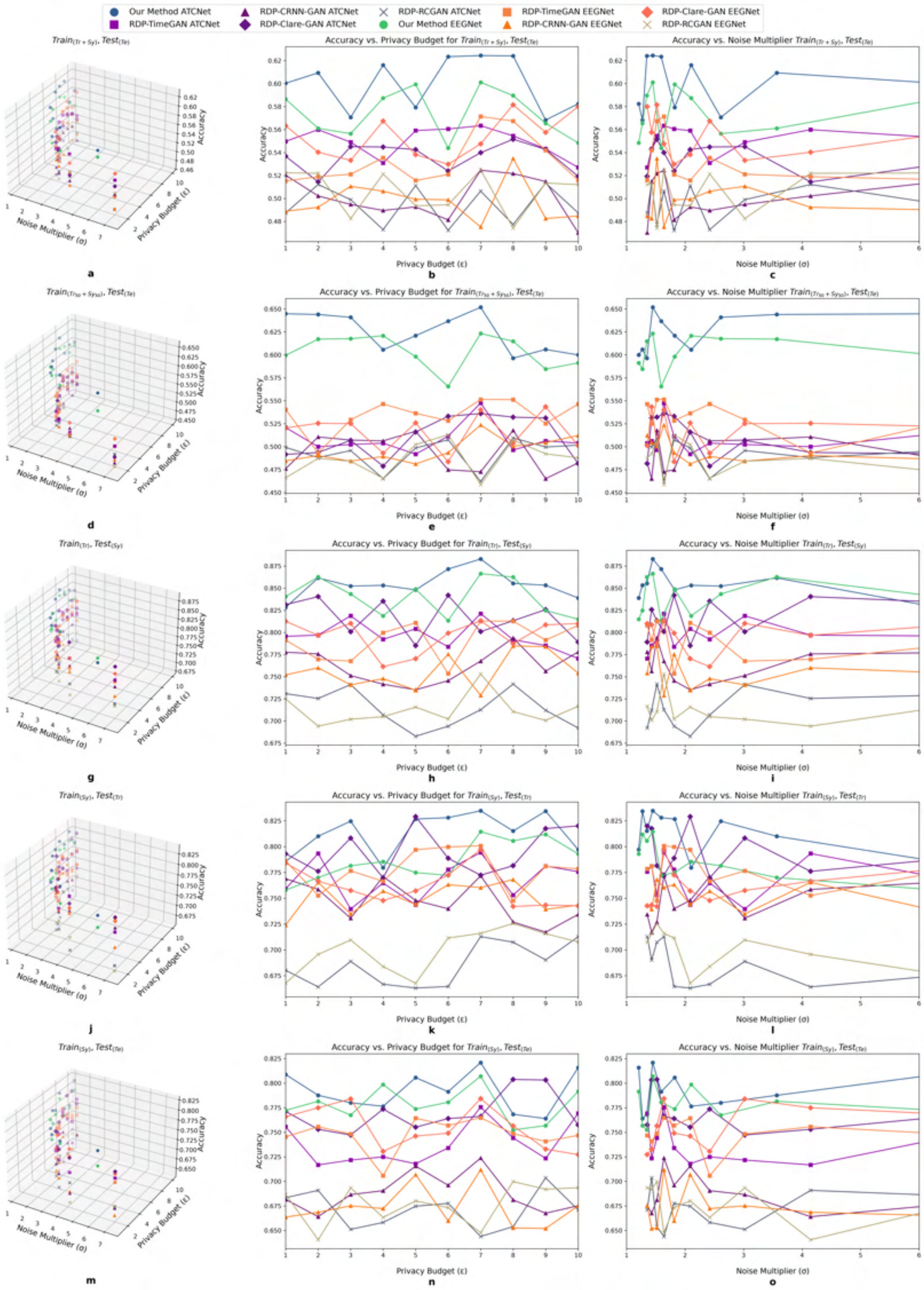

Figure S1. Comparison of different models under various privacy budgets and noise multipliers for subject A2.

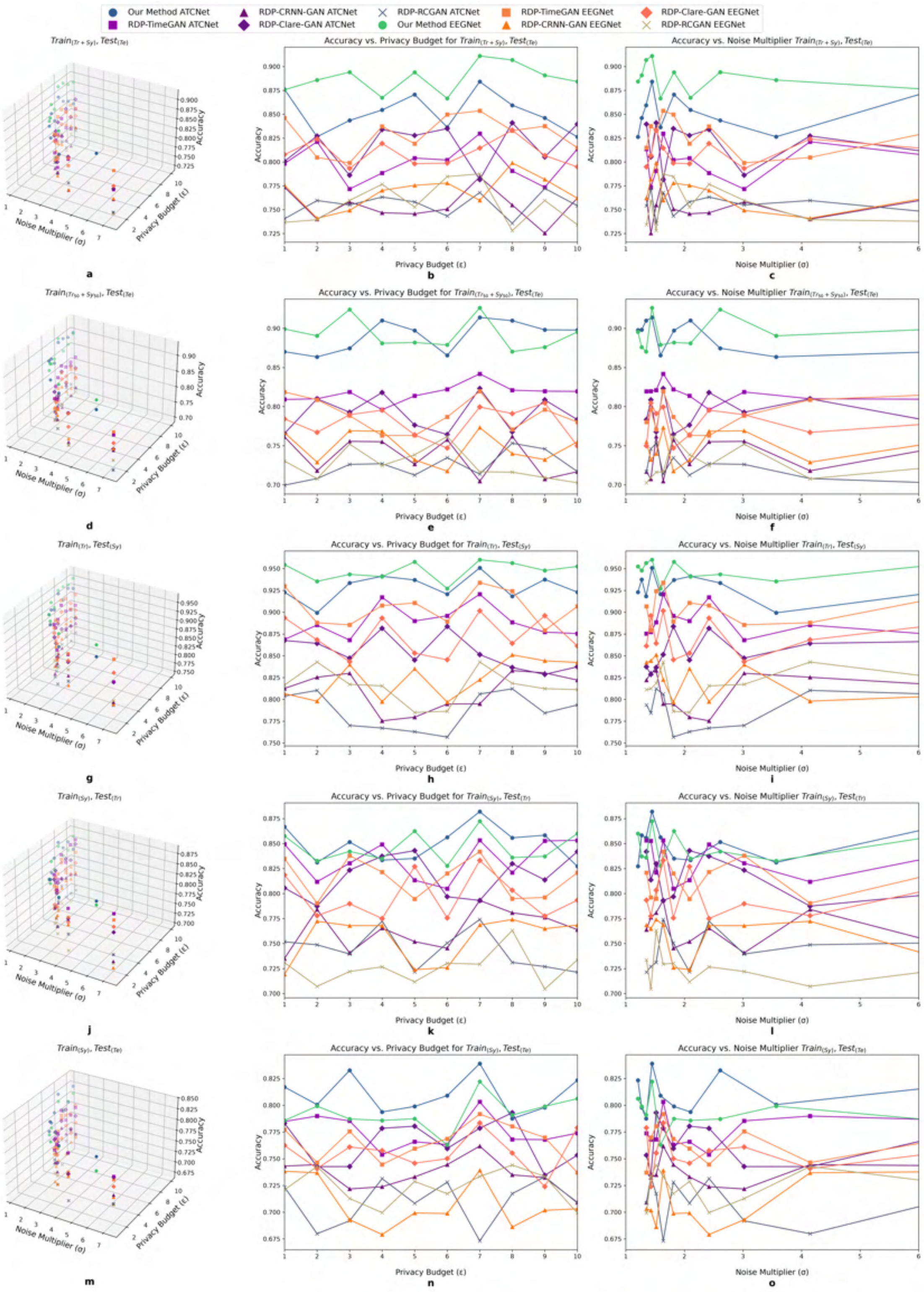

Figure S2. Comparison of different models under various privacy budgets and noise multipliers for subject A3.

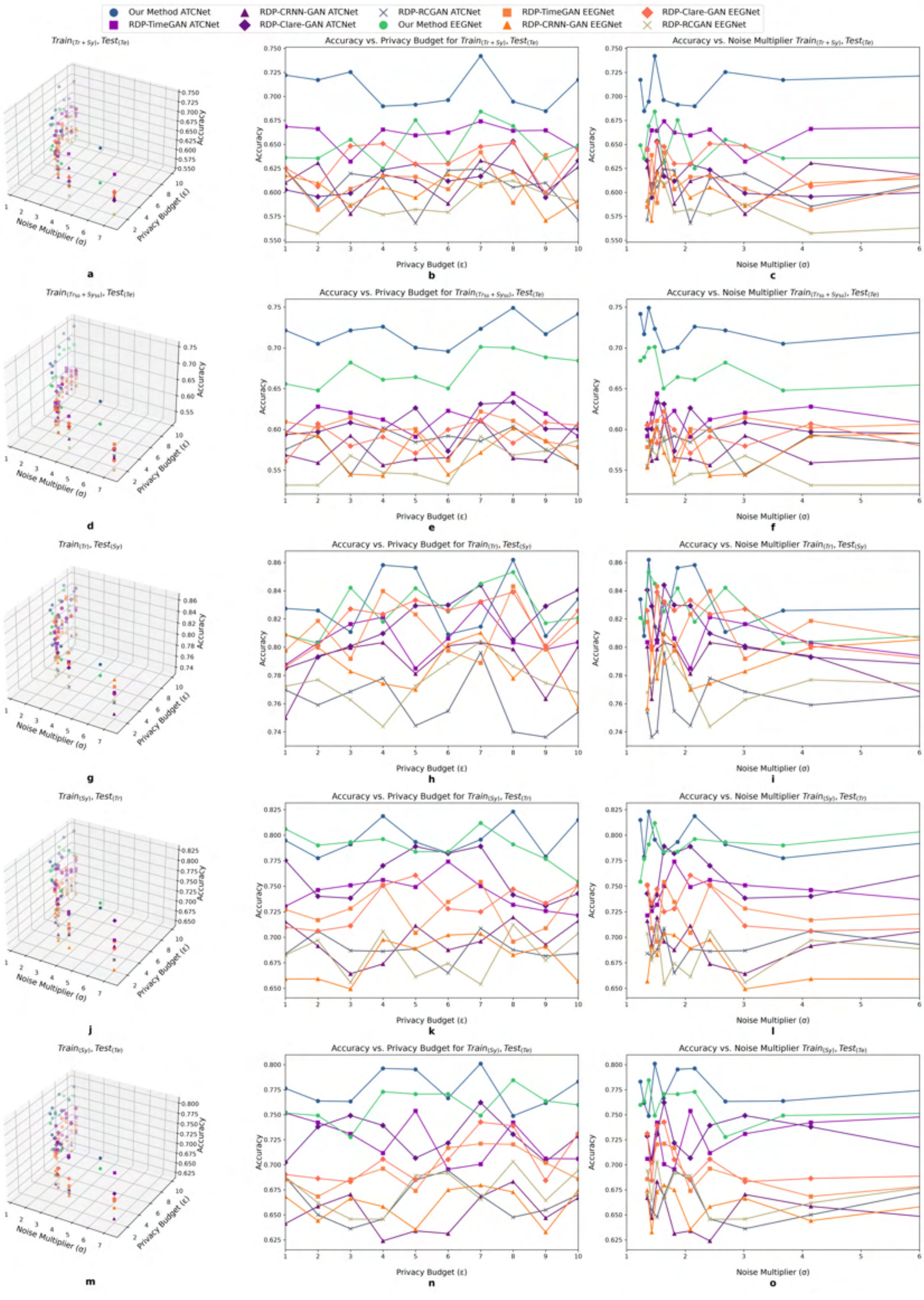

Figure S3. Comparison of different models under various privacy budgets and noise multipliers for subject A4.

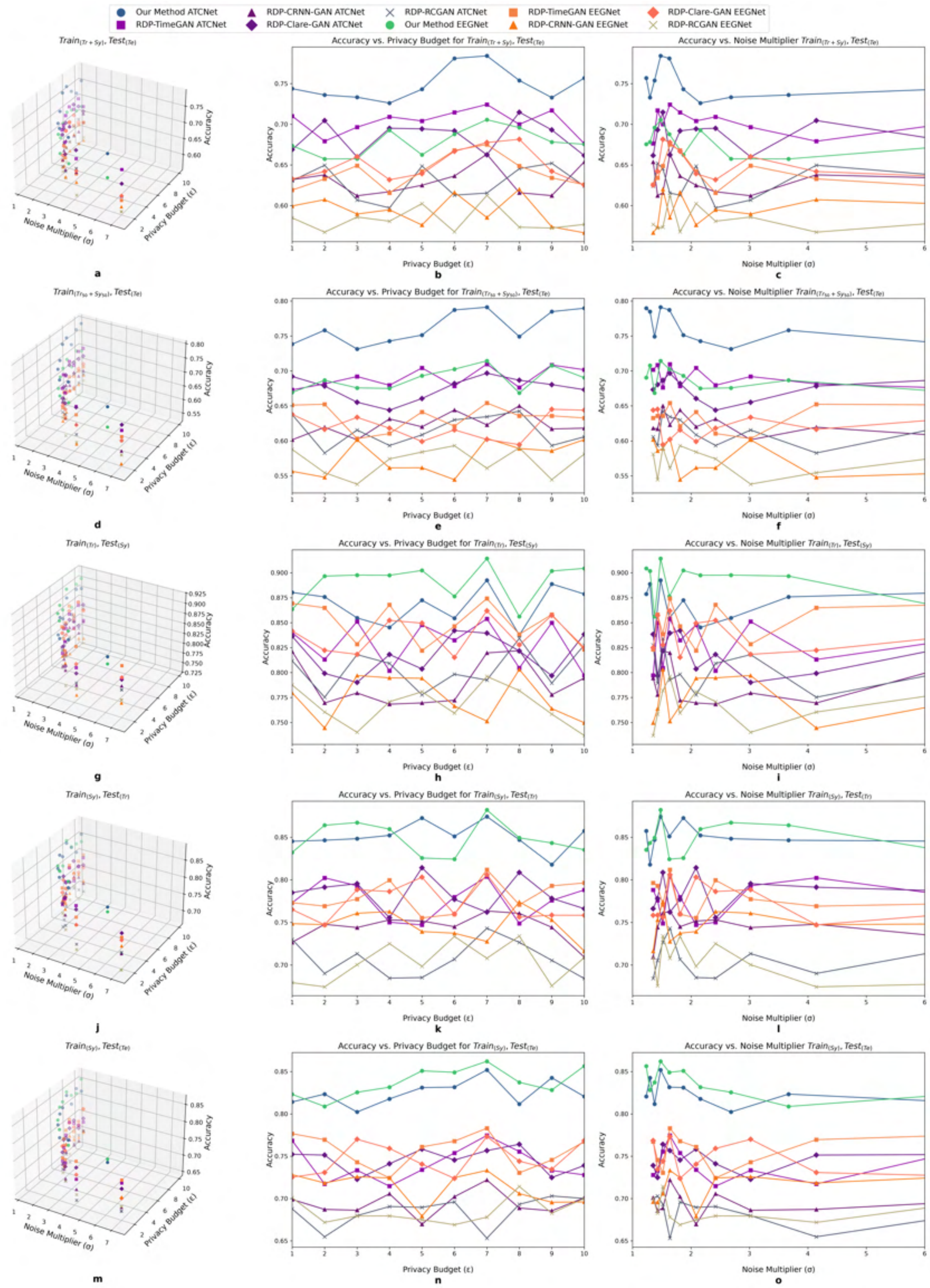

Figure S4. Comparison of different models under various privacy budgets and noise multipliers for subject A5.

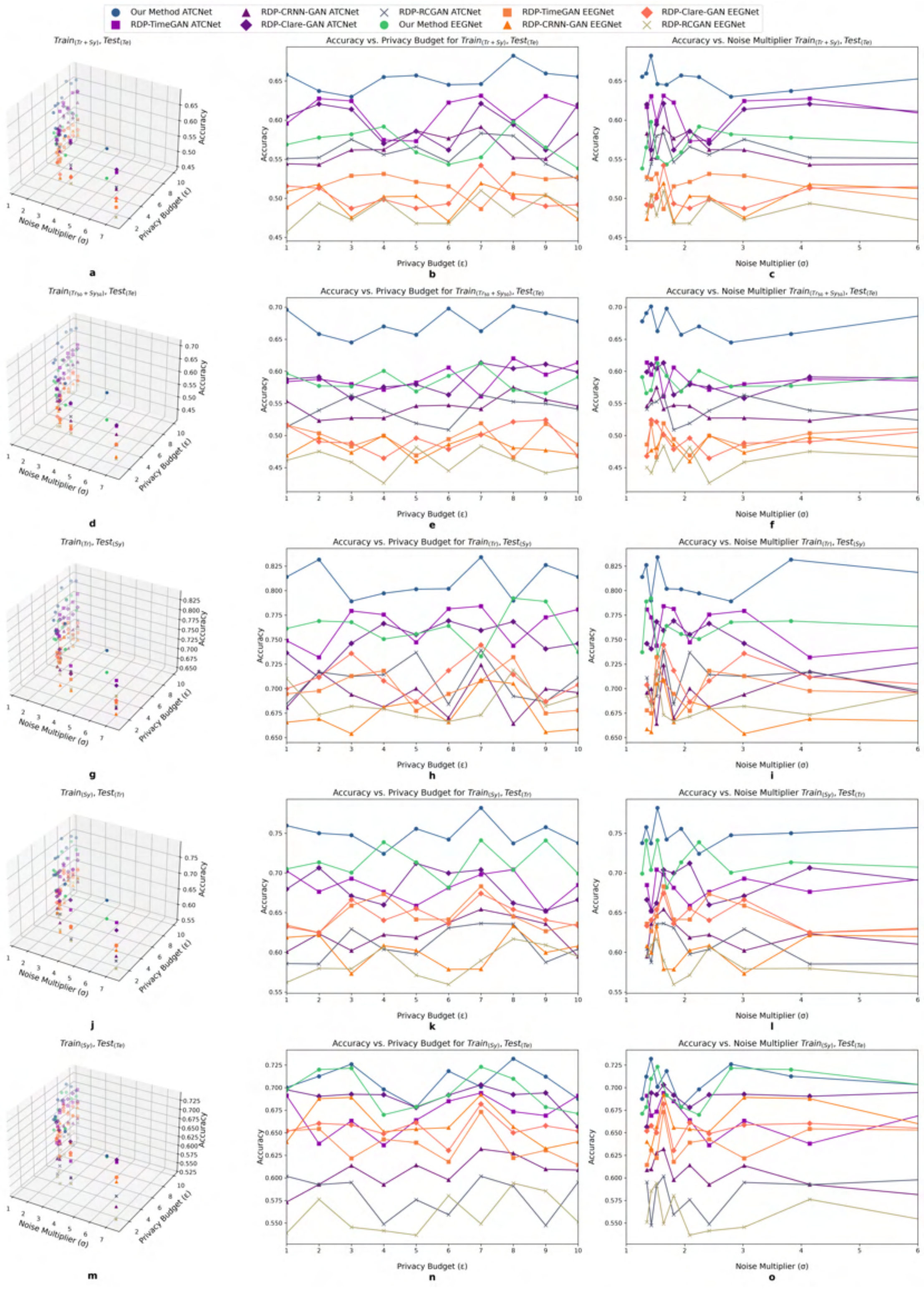

Figure S5. Comparison of different models under various privacy budgets and noise multipliers for subject A6.

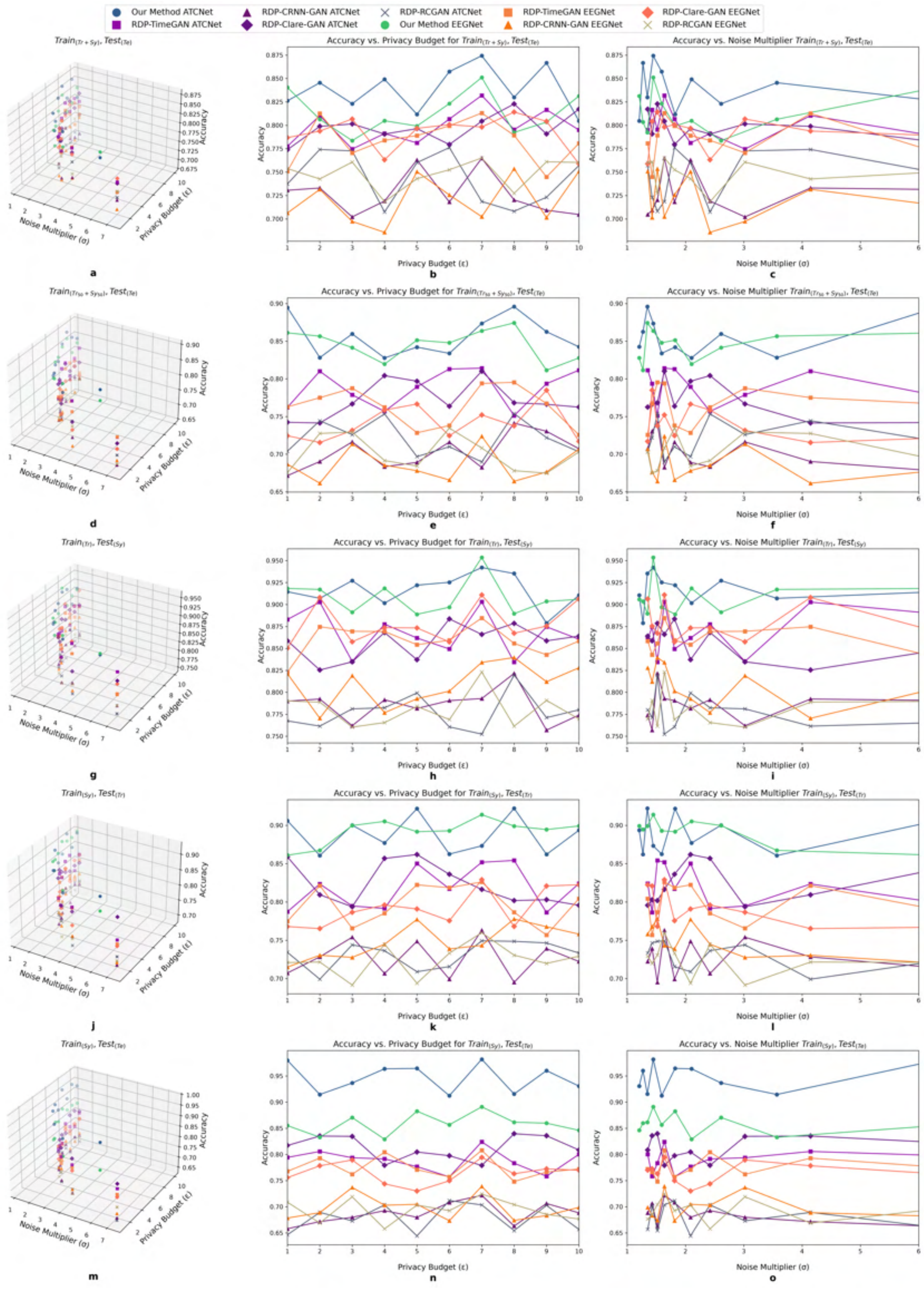

Figure S6. Comparison of different models under various privacy budgets and noise multipliers for subject A7.

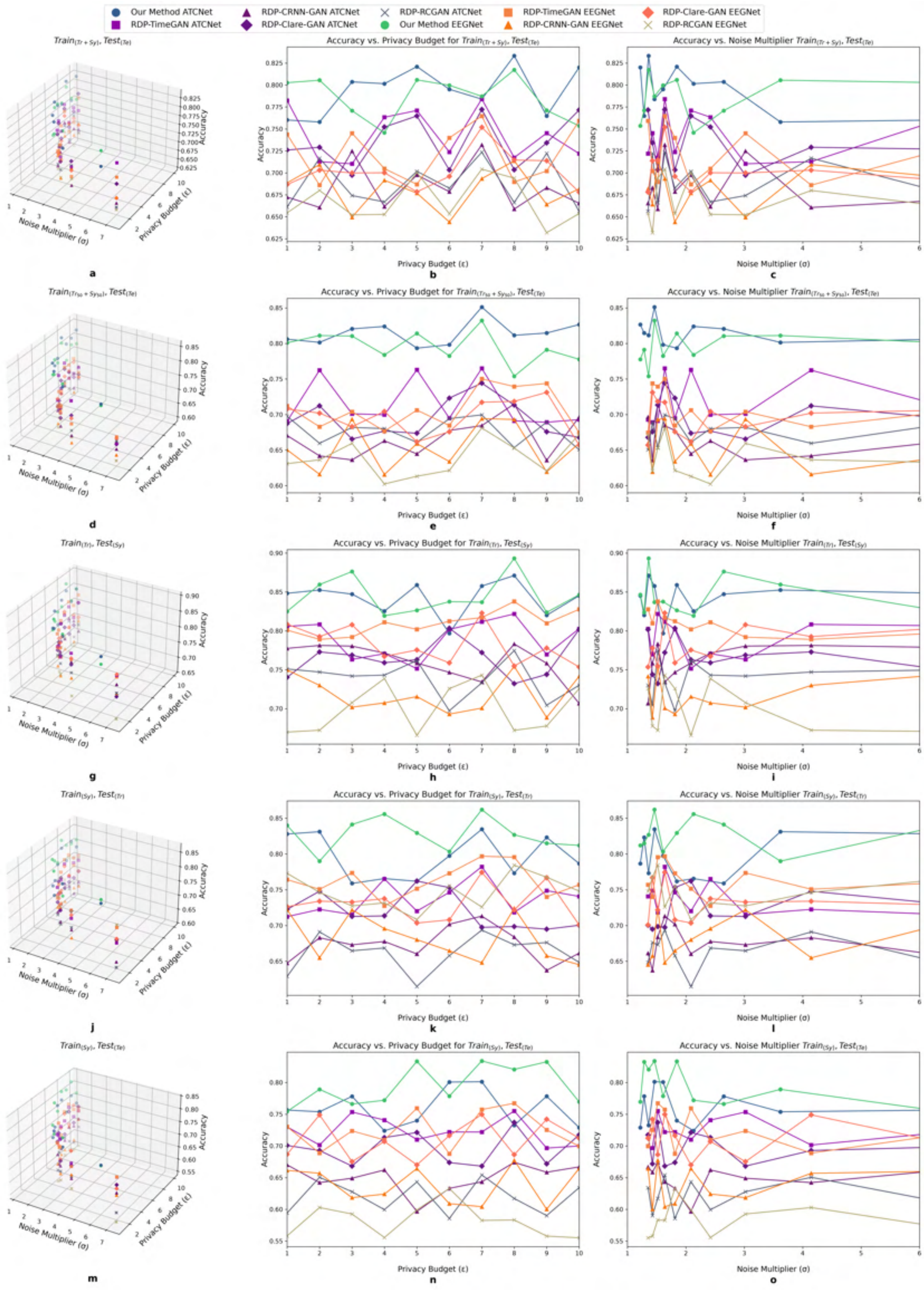

Figure S7. Comparison of different models under various privacy budgets and noise multipliers for subject A8.

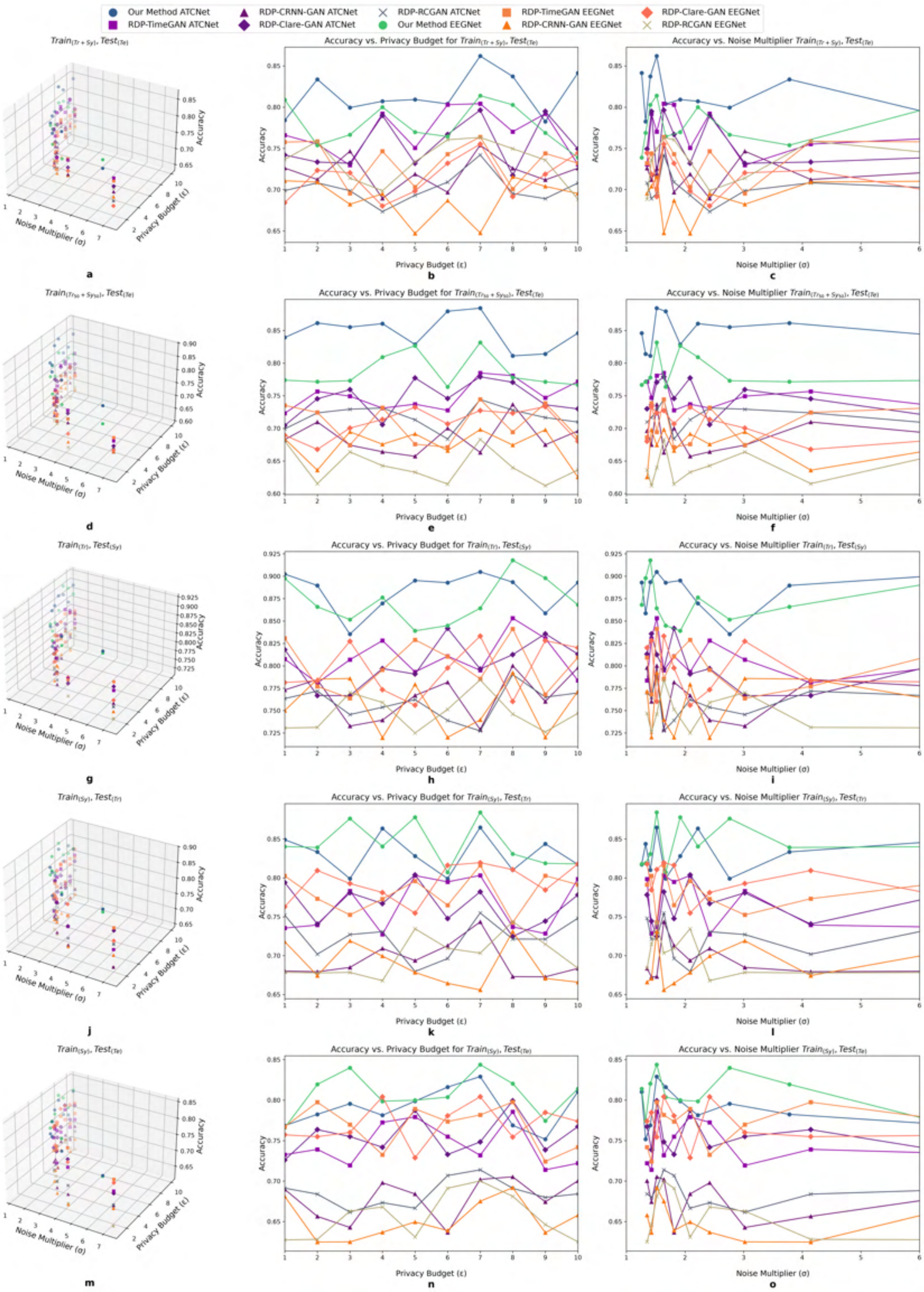

Figure S8. Comparison of different models under various privacy budgets and noise multipliers for subject A9.

2 FULL BLACK-BOX ATTACK RESULTS:

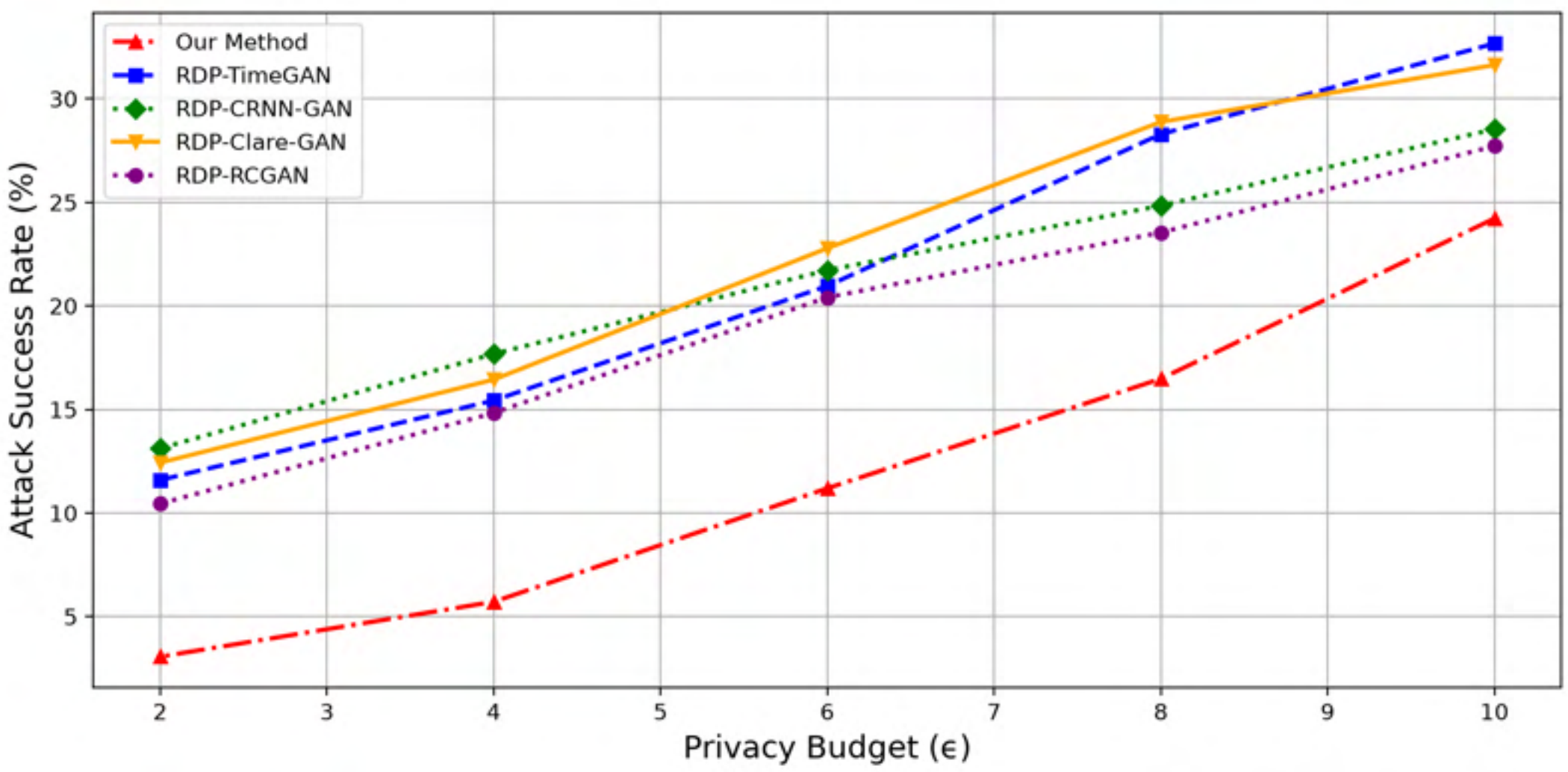

Figure S9. Attack success rate (%) for subject A2 across different ϵ values. A higher percentage signifies a more successful attack, whereas a lower percentage indicates better privacy protection.

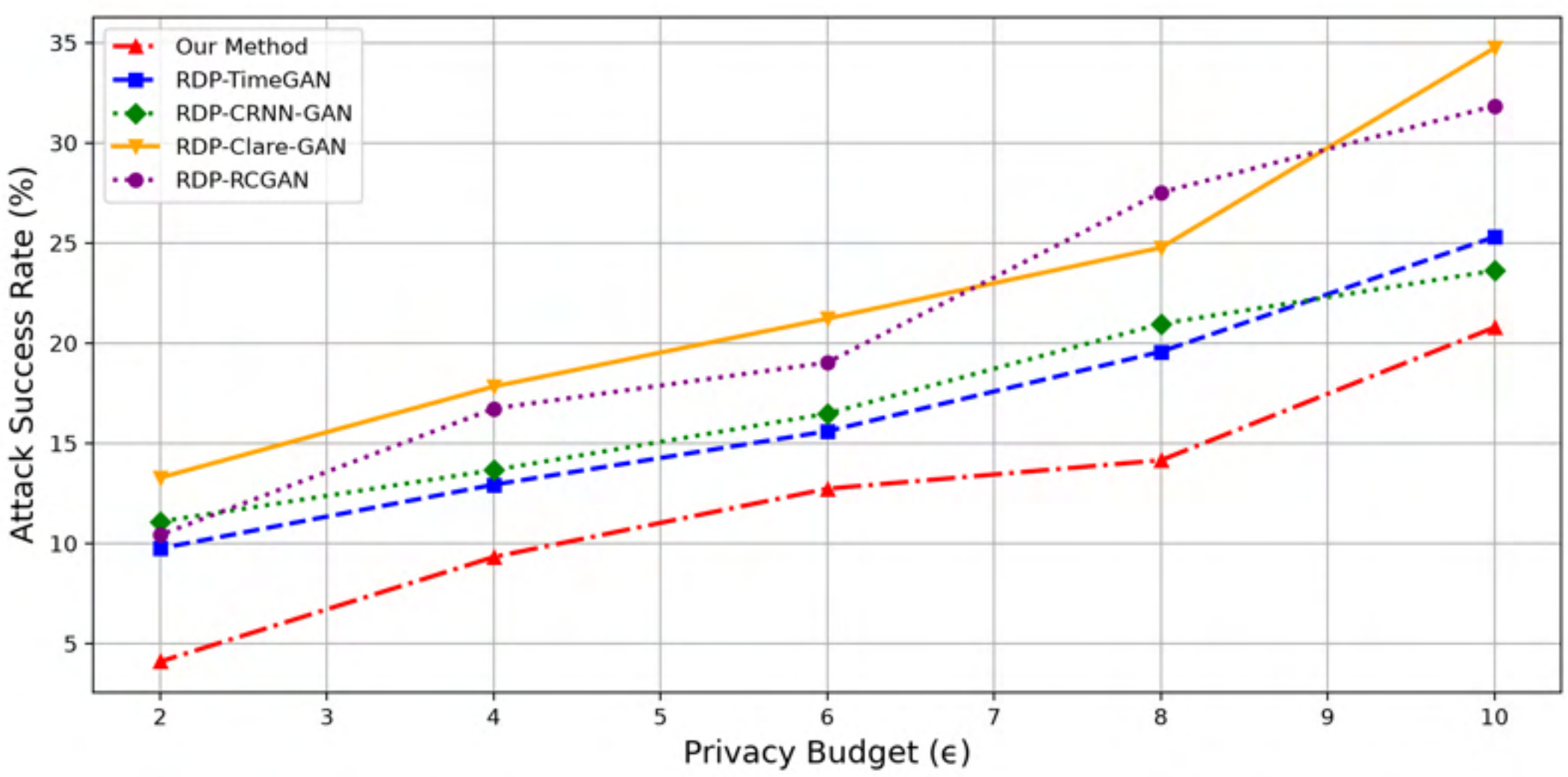

Figure S10. Attack success rate (%) for subject A3 across different ϵ values. A higher percentage signifies a more successful attack, whereas a lower percentage indicates better privacy protection.

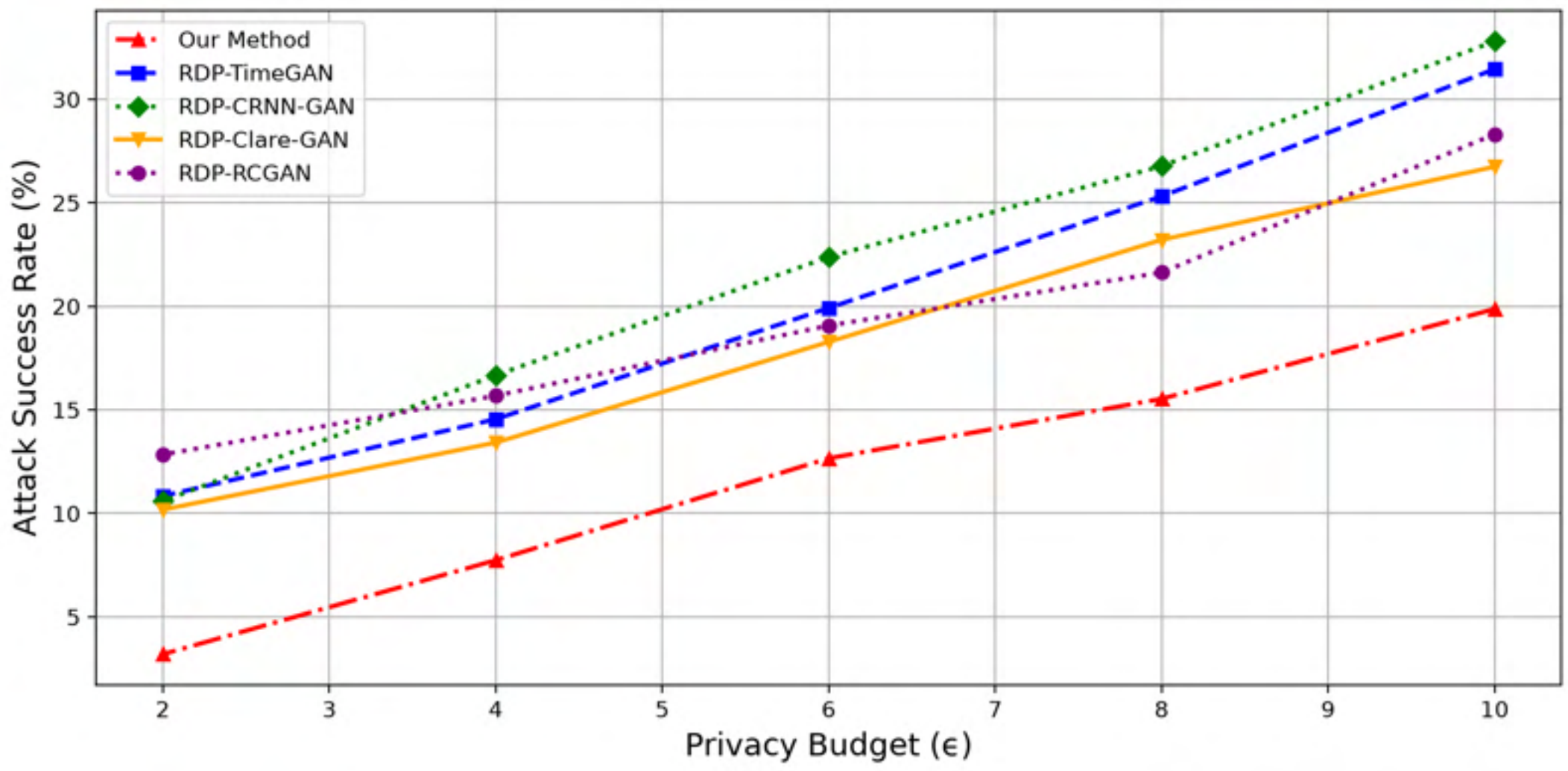

Figure S11. Attack success rate (%) for subject A4 across different ϵ values. A higher percentage signifies a more successful attack, whereas a lower percentage indicates better privacy protection.

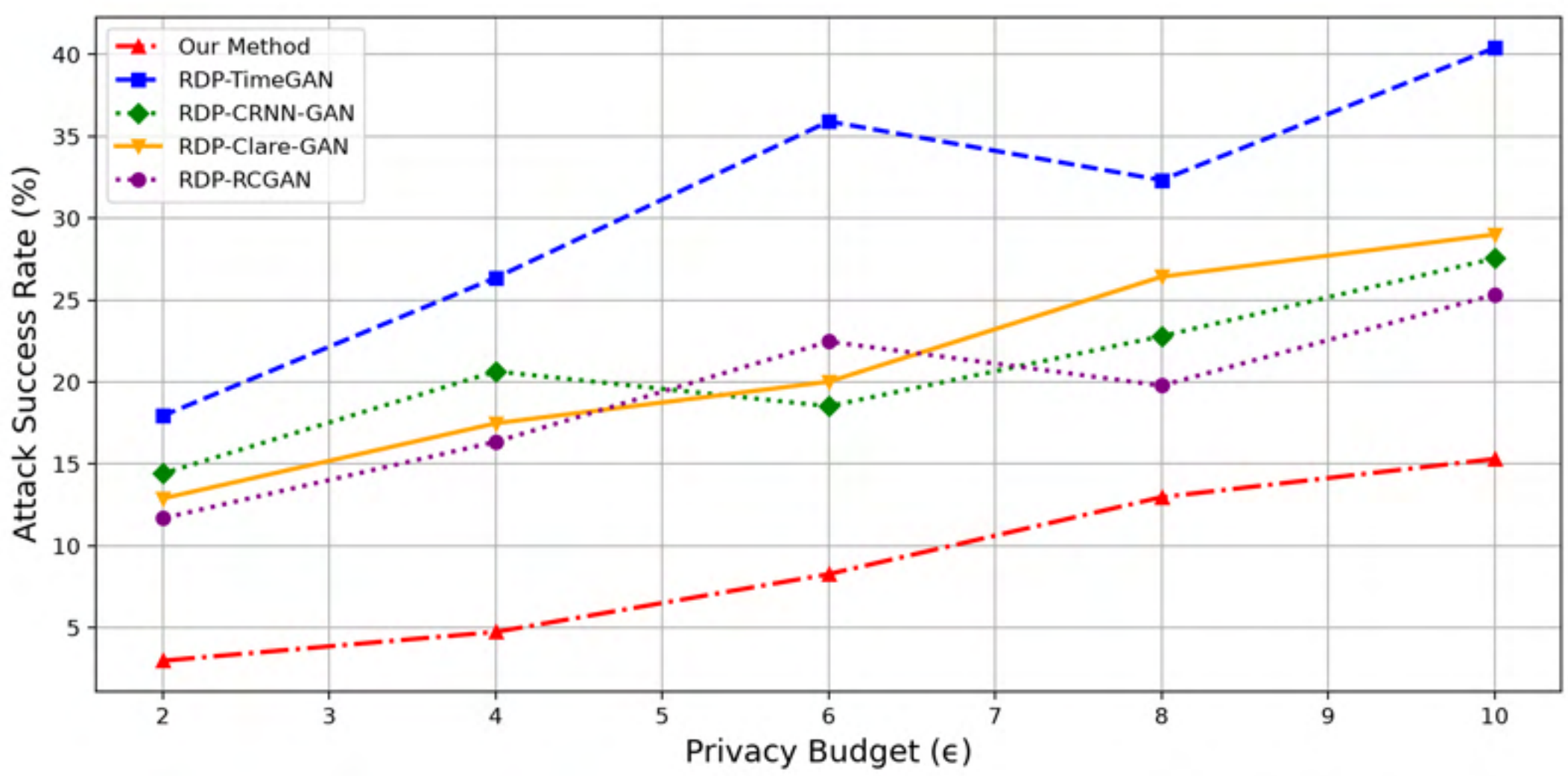

Figure S12. Attack success rate (%) for subject A5 across different ϵ values. A higher percentage signifies a more successful attack, whereas a lower percentage indicates better privacy protection.

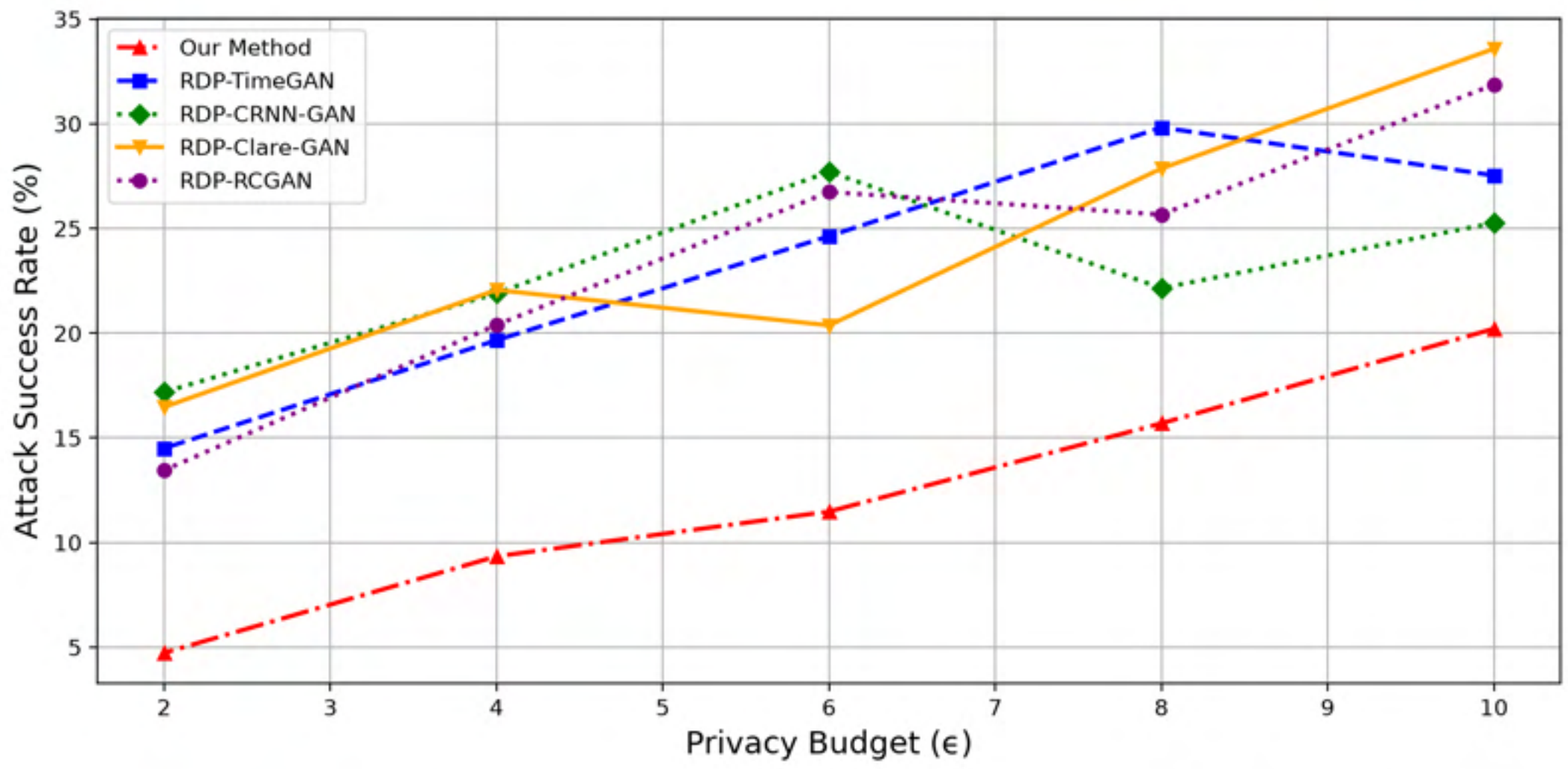

Figure S13. Attack success rate (%) for subject A6 across different ϵ values. A higher percentage signifies a more successful attack, whereas a lower percentage indicates better privacy protection.

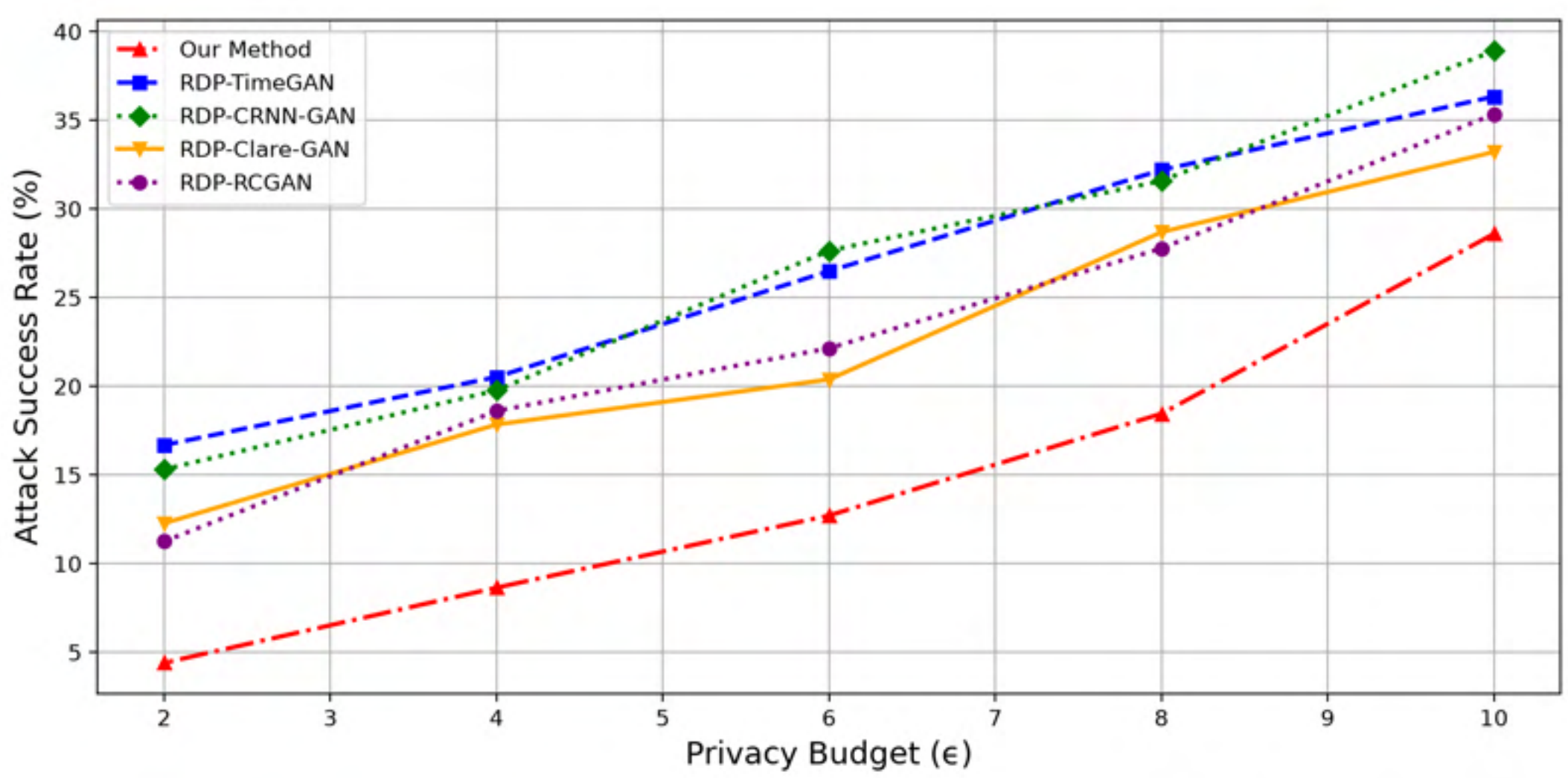

Figure S14. Attack success rate (%) for subject A7 across different ϵ values. A higher percentage signifies a more successful attack, whereas a lower percentage indicates better privacy protection.

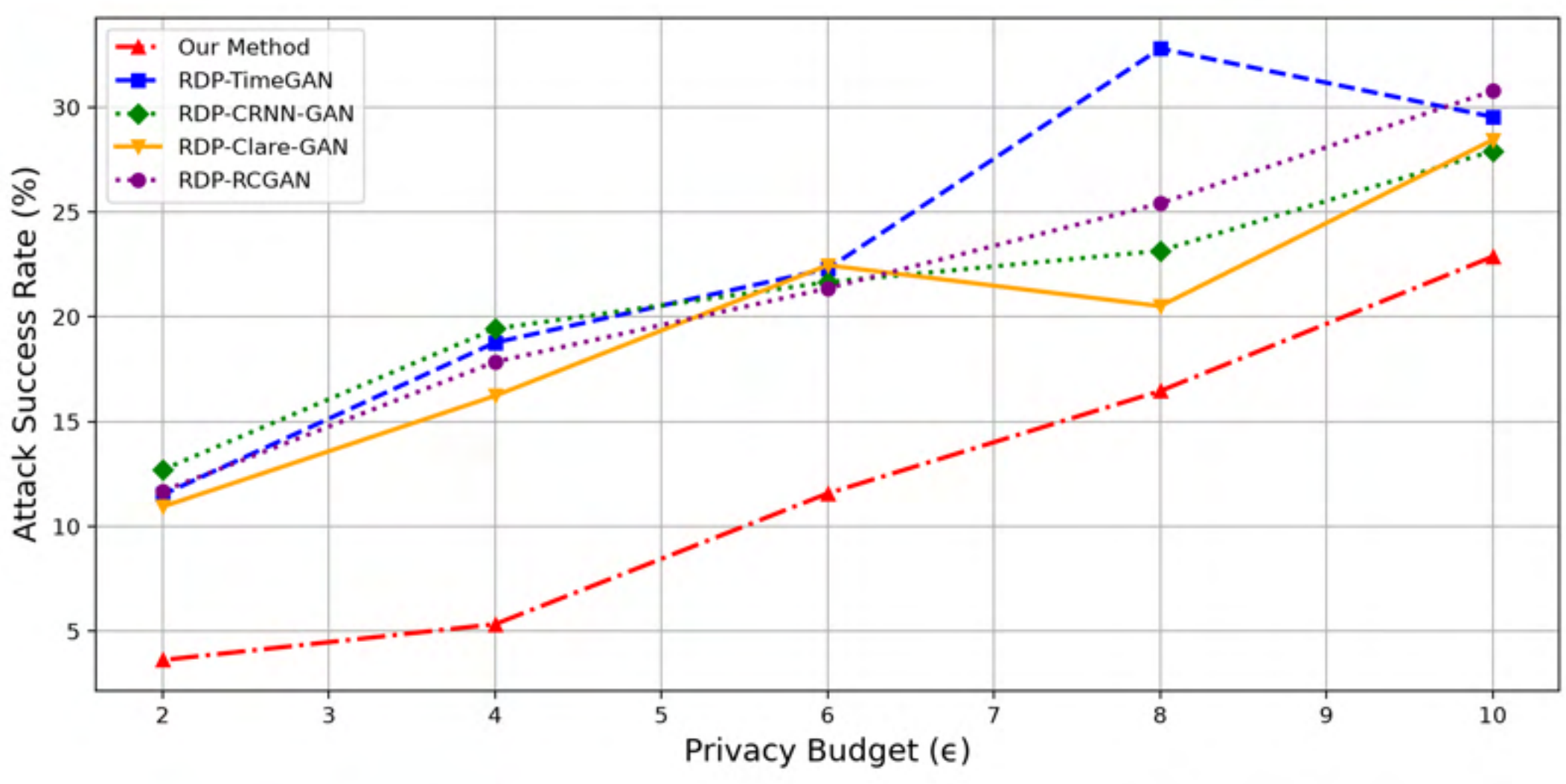

Figure S15. Attack success rate (%) for subject A8 across different ϵ values. A higher percentage signifies a more successful attack, whereas a lower percentage indicates better privacy protection.

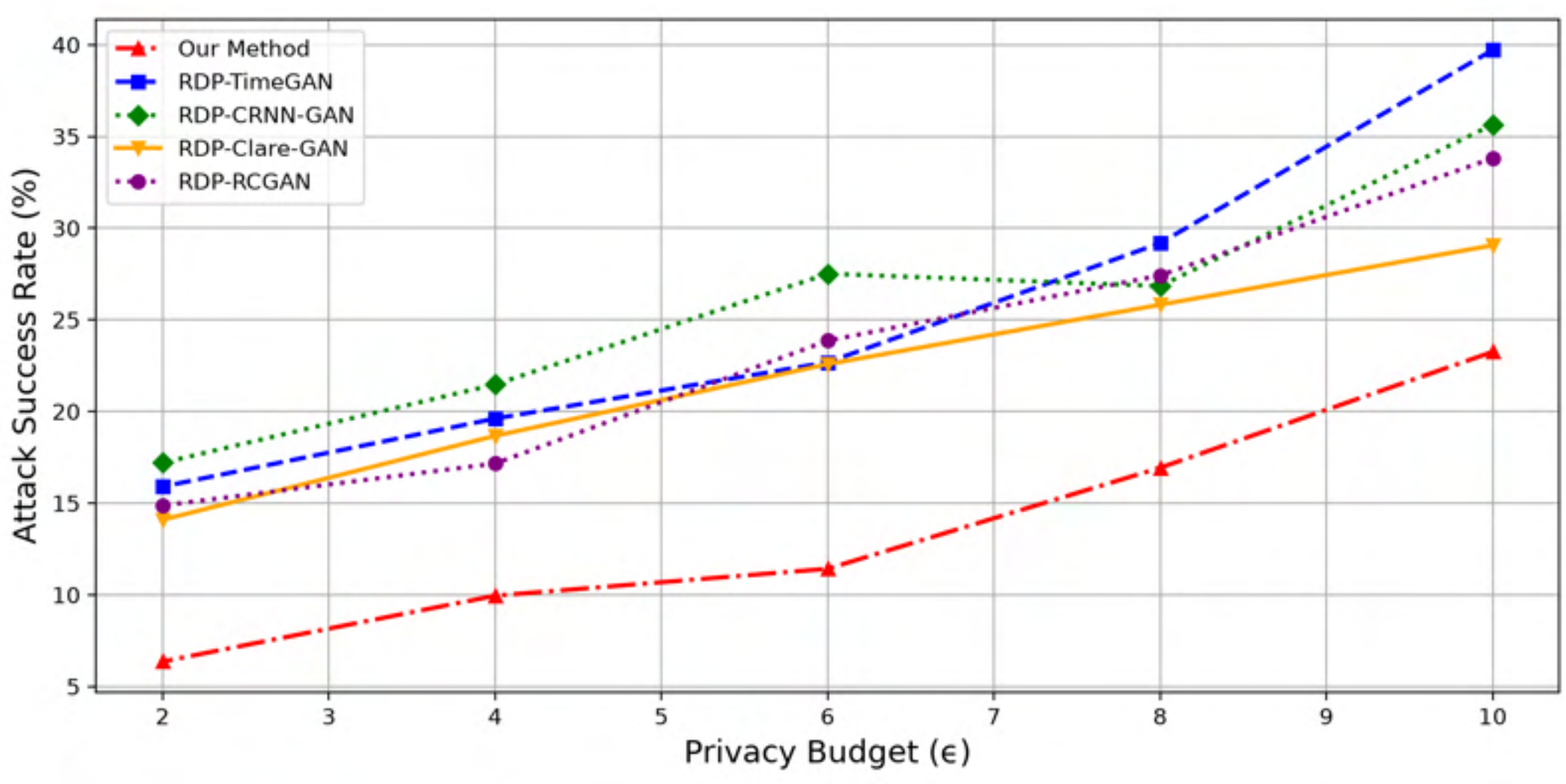

Figure S16. Attack success rate (%) for subject A9 across different ϵ values. A higher percentage signifies a more successful attack, whereas a lower percentage indicates better privacy protection.

3 VISUALIZATION OF HIGH-DIMENSIONAL EEG DATA USING 3D T-SNE:

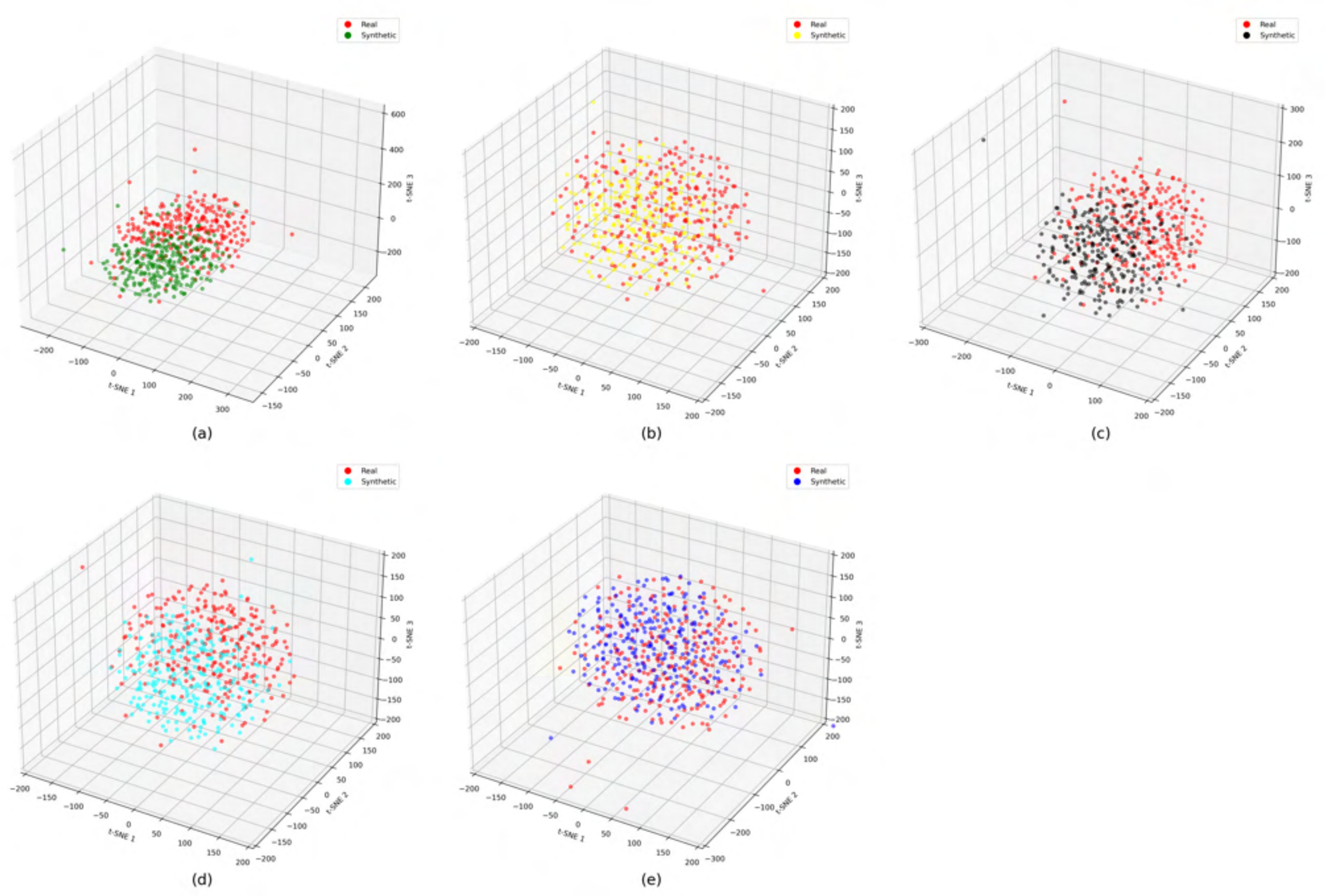

Figure S17. 3D t-SNE visualization of high-dimensional EEG data for subject A2. Each point in red color represents a real EEG data sample, and other synthetic data color-coded by the model used for generation. (a) Real data vs. proposed Spiking GAN (b) Real data vs. RDP-TimeGAN (c) Real data vs. RDP-CRNN-GAN (d) Real data vs. RDP-Clare-GAN and (e) Real data vs. RDP-RCGAN.

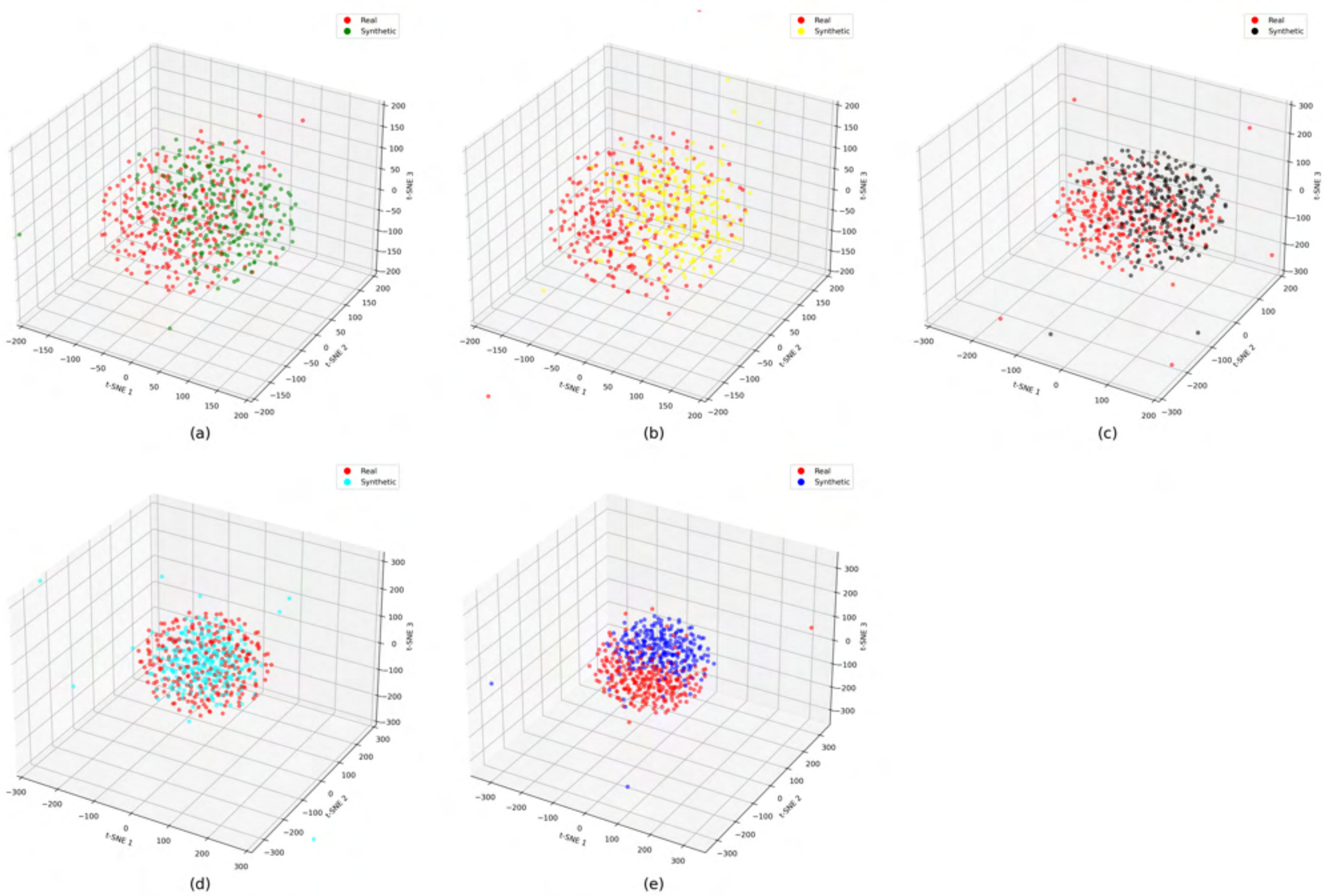

Figure S18. 3D t-SNE visualization of high-dimensional EEG data for subject A3. Each point in red color represents a real EEG data sample, and other synthetic data color-coded by the model used for generation. (a) Real data vs. proposed Spiking GAN (b) Real data vs. RDP-TimeGAN (c) Real data vs. RDP-CRNN-GAN (d) Real data vs. RDP-Clare-GAN and (e) Real data vs. RDP-RCGAN.

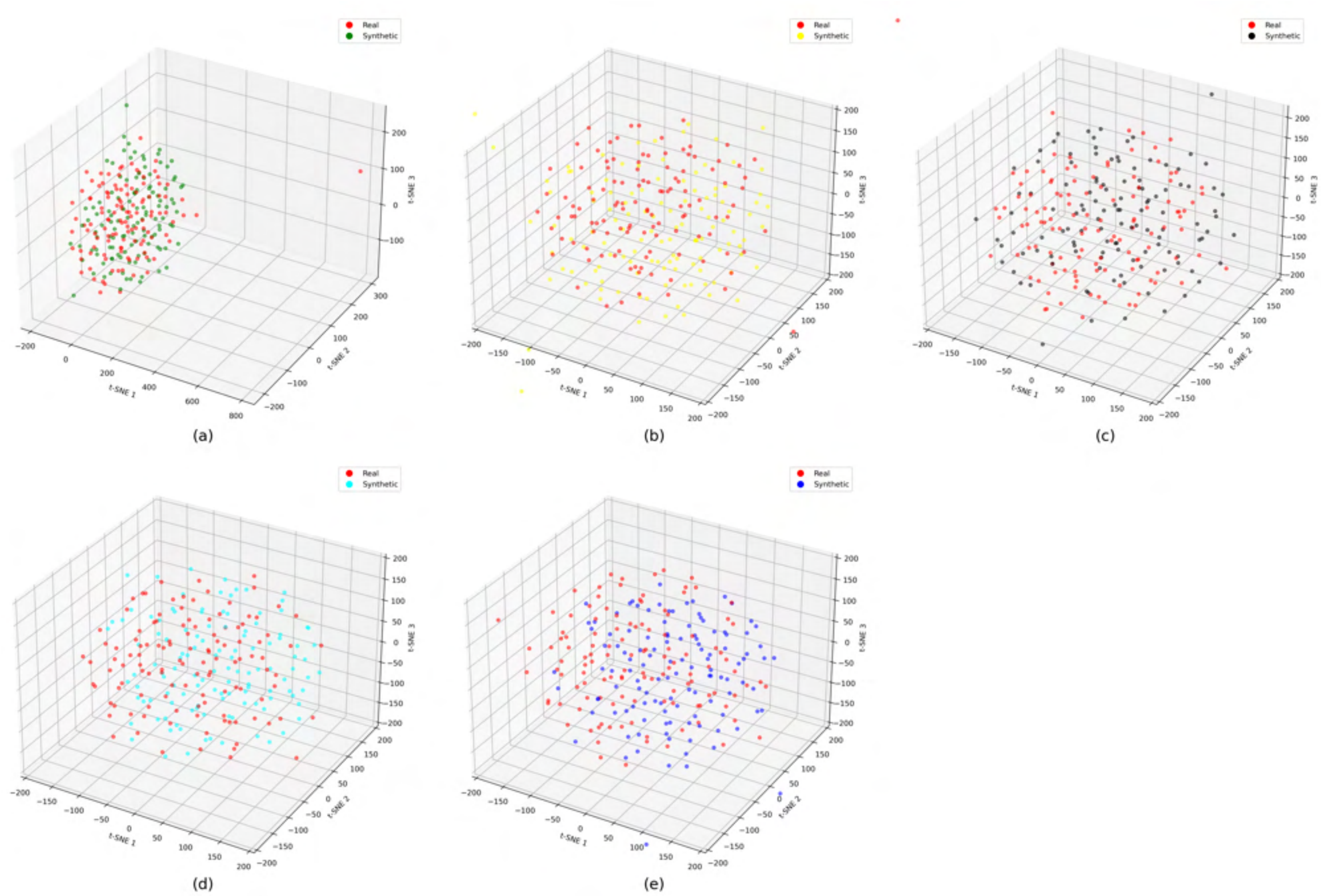

Figure S19. 3D t-SNE visualization of high-dimensional EEG data for subject A4. Each point in red color represents a real EEG data sample, and other synthetic data color-coded by the model used for generation. (a) Real data vs. proposed Spiking GAN (b) Real data vs. RDP-TimeGAN (c) Real data vs. RDP-CRNN-GAN (d) Real data vs. RDP-Clare-GAN and (e) Real data vs. RDP-RCGAN.

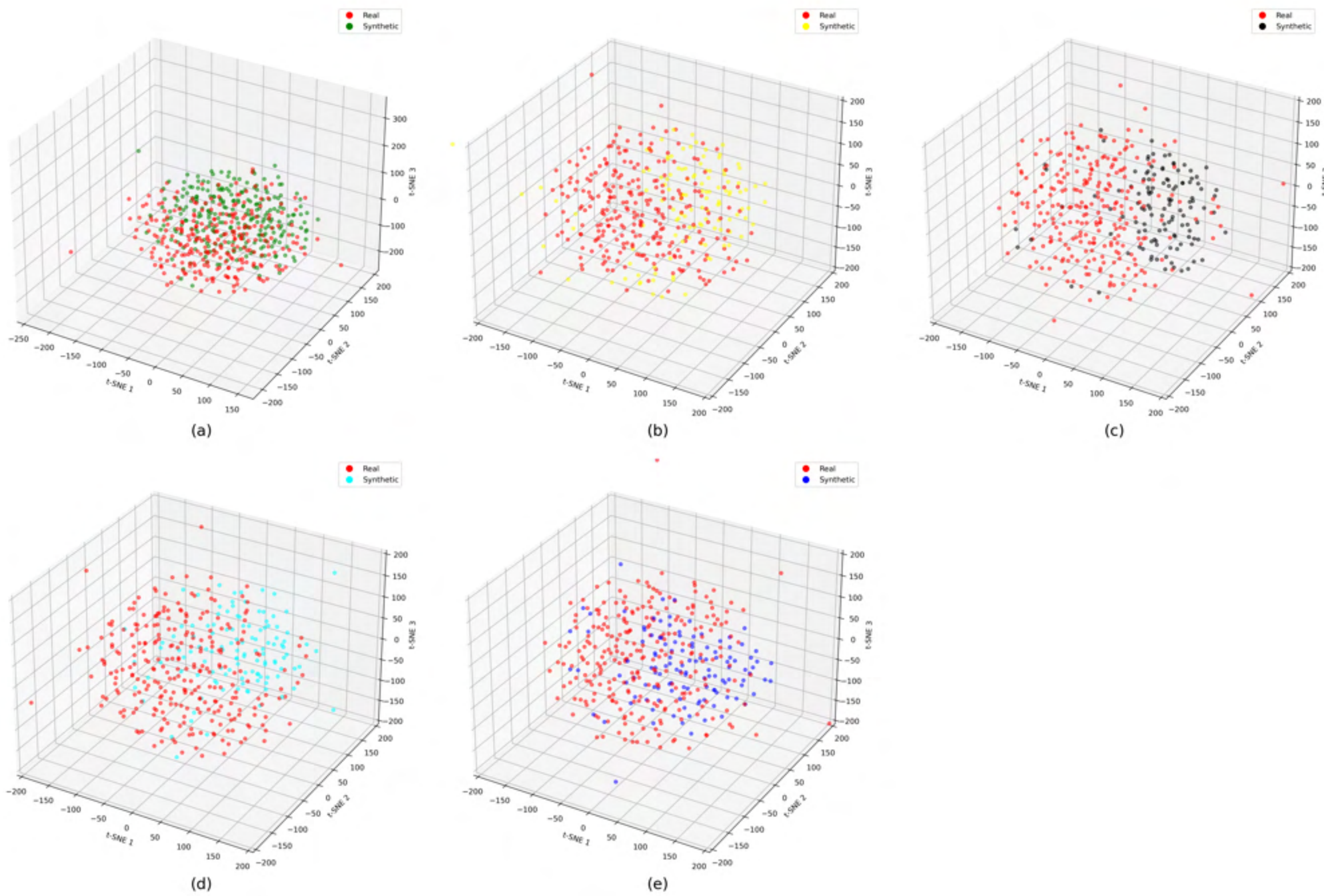

Figure S20. 3D t-SNE visualization of high-dimensional EEG data for subject A5. Each point in red color represents a real EEG data sample, and other synthetic data color-coded by the model used for generation. (a) Real data vs. proposed Spiking GAN (b) Real data vs. RDP-TimeGAN (c) Real data vs. RDP-CRNN-GAN (d) Real data vs. RDP-Clare-GAN and (e) Real data vs. RDP-RCGAN.

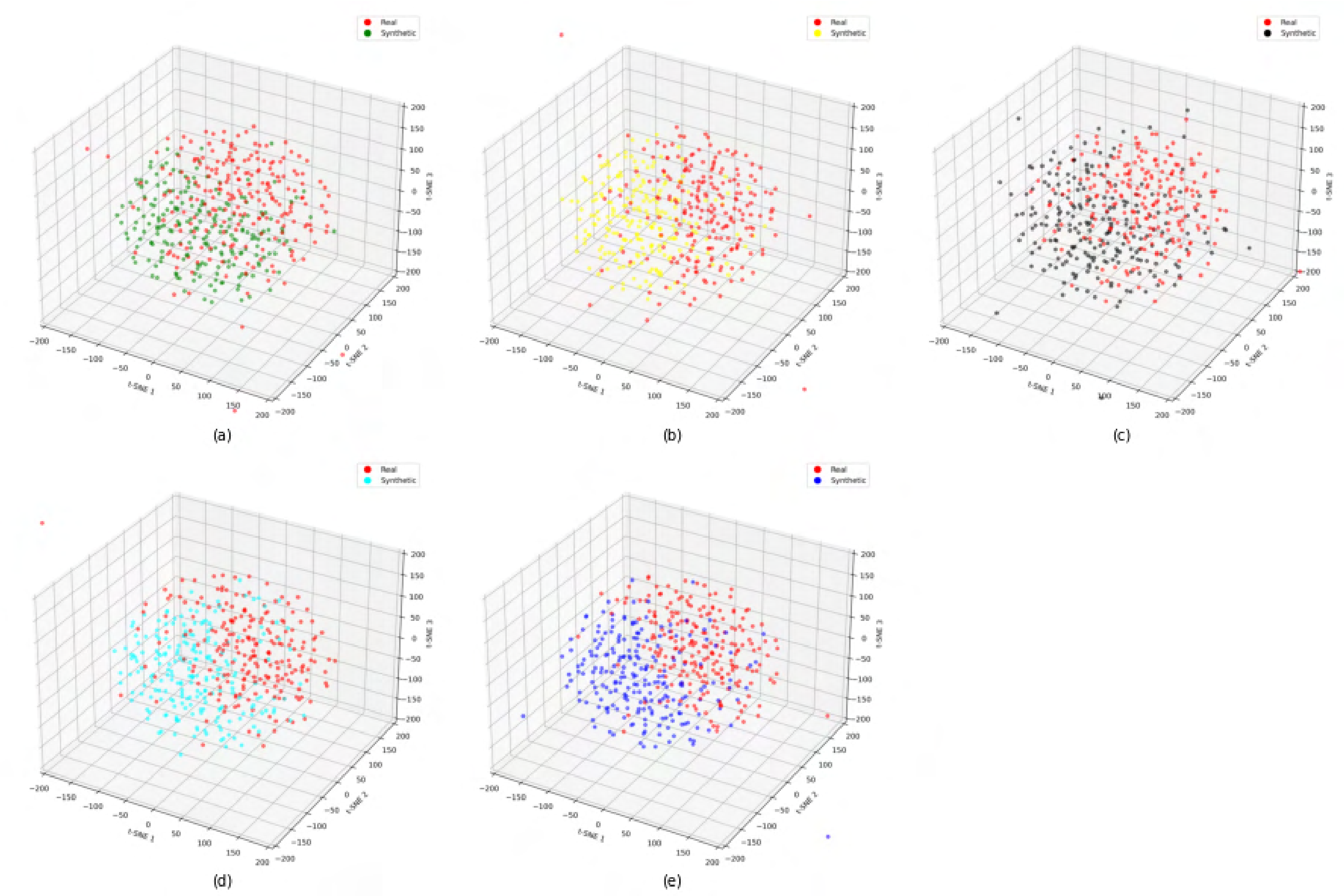

Figure S21. 3D t-SNE visualization of high-dimensional EEG data for subject A6. Each point in red color represents a real EEG data sample, and other synthetic data color-coded by the model used for generation. (a) Real data vs. proposed Spiking GAN (b) Real data vs. RDP-TimeGAN (c) Real data vs. RDP-CRNN-GAN (d) Real data vs. RDP-Clare-GAN and (e) Real data vs. RDP-RCGAN.

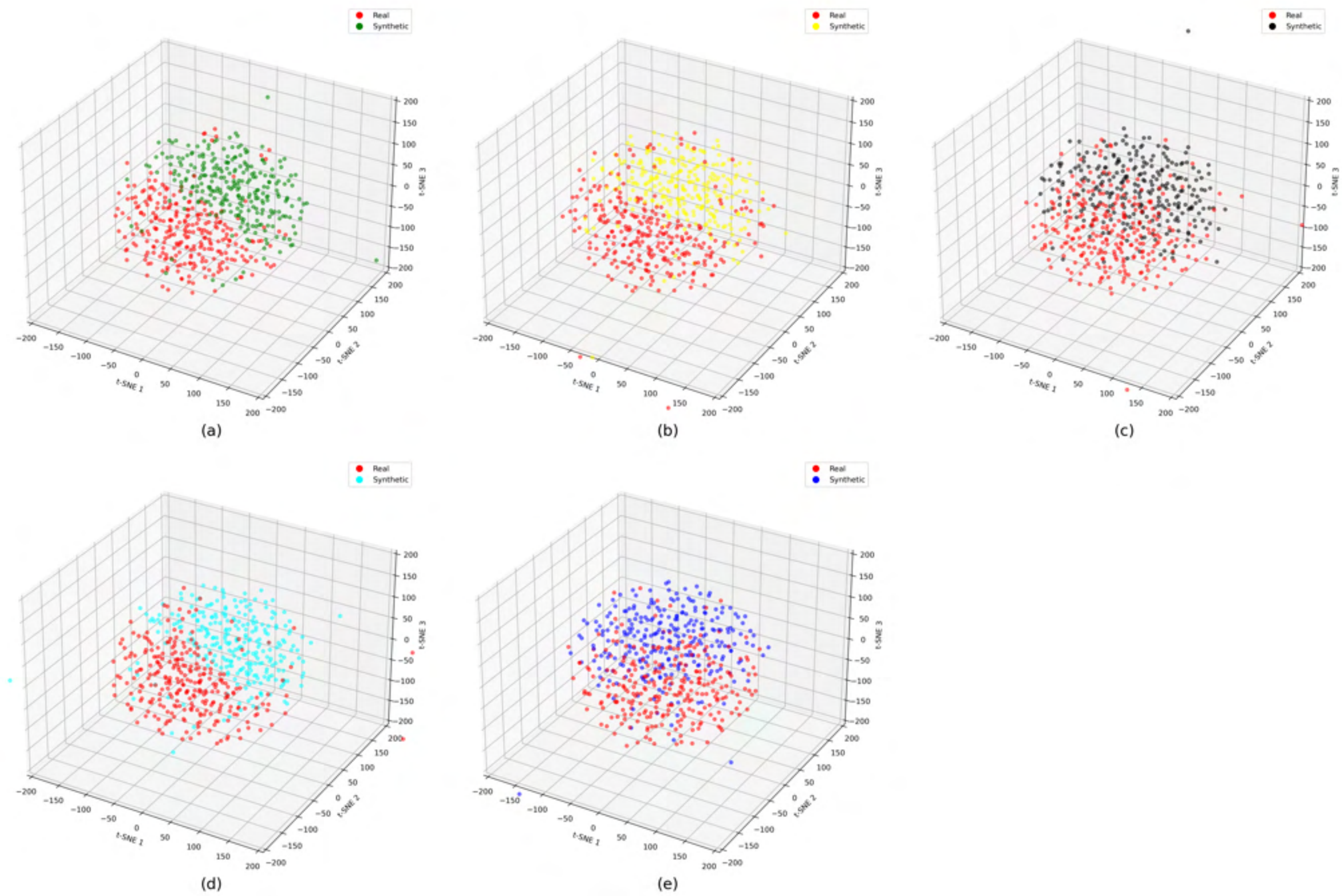

Figure S22. 3D t-SNE visualization of high-dimensional EEG data for subject A7. Each point in red color represents a real EEG data sample, and other synthetic data color-coded by the model used for generation. (a) Real data vs. proposed Spiking GAN (b) Real data vs. RDP-TimeGAN (c) Real data vs. RDP-CRNN-GAN (d) Real data vs. RDP-Clare-GAN and (e) Real data vs. RDP-RCGAN.

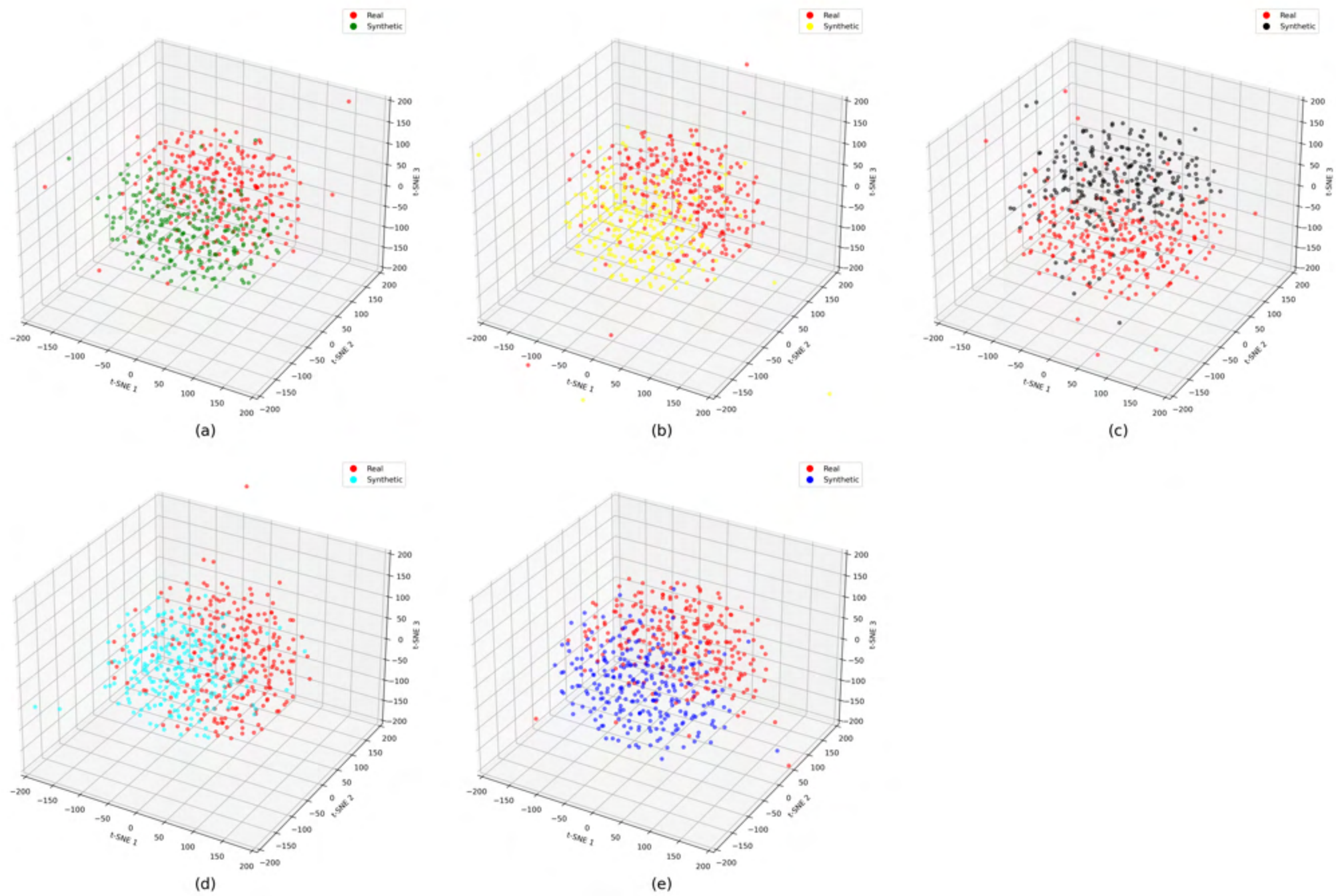

Figure S23. 3D t-SNE visualization of high-dimensional EEG data for subject A8. Each point in red color represents a real EEG data sample, and other synthetic data color-coded by the model used for generation. (a) Real data vs. proposed Spiking GAN (b) Real data vs. RDP-TimeGAN (c) Real data vs. RDP-CRNN-GAN (d) Real data vs. RDP-Clare-GAN and (e) Real data vs. RDP-RCGAN.

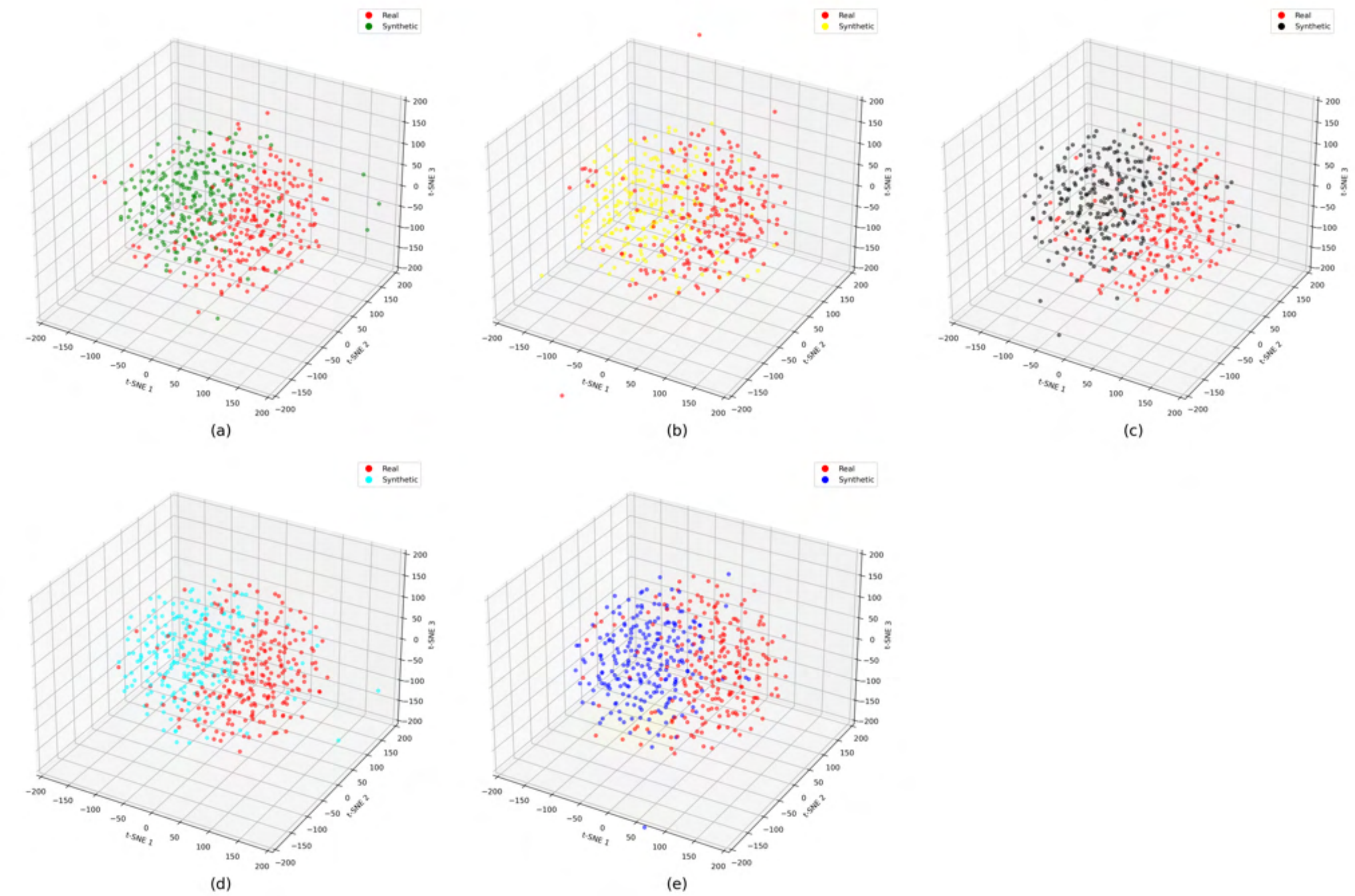

Figure S24. 3D t-SNE visualization of high-dimensional EEG data for subject A9. Each point in red color represents a real EEG data sample, and other synthetic data color-coded by the model used for generation. (a) Real data vs. proposed Spiking GAN (b) Real data vs. RDP-TimeGAN (c) Real data vs. RDP-CRNN-GAN (d) Real data vs. RDP-Clare-GAN and (e) Real data vs. RDP-RCGAN.
